# Effects of chronic consumption of specific fruit (berries, citrus and cherries) on CVD risk factors: a systematic review and meta-analysis of randomised controlled trials

**DOI:** 10.1007/s00394-020-02299-w

**Published:** 2020-06-13

**Authors:** Yueyue Wang, Jose Lara Gallegos, Crystal Haskell-Ramsay, John K. Lodge

**Affiliations:** 1 Department of Applied Sciences, Faculty of Health and Life Sciences, Northumbria University, EBD223 Ellison Building, Newcastle upon Tyne, NE1 8ST UK; 2 Department of Psychology, Faculty of Health and Life Sciences, Northumbria University, Newcastle-upon-Tyne, UK

**Keywords:** Fruit, Intervention, Endothelial function, CVD risk factors, Systematic review, Meta-analysis

## Abstract

**Purpose:**

This review aims to compare the magnitude of the effects of chronic consumption of fruits; specifically berries, citrus and cherries on cardiovascular disease (CVD) risk factors.

**Methods:**

PubMed, Web of Science, Scopus, and psycARTICLES were searched from inception until January 2020. Forty-five chronic (≥ 1 week) randomised controlled trials assessing CVD risk factors including endothelial (dys)function, blood pressure (BP), blood lipids and inflammatory biomarkers were included.

**Results:**

Investigated interventions reported improvements in endothelial function (*n* = 8), inflammatory biomarkers and lipid status (*n* = 14), and BP (*n* = 10). Berries including juice of barberry, cranberry, grape, pomegranate, powder of blueberry, grape, raspberry and freeze-dried strawberry significantly reduced SBP by 3.68 mmHg (95% CI − 6.79 to − 0.58; *P *= 0.02) and DBP by 1.52 mmHg (95% CI − 2.87 to − 0.18, *P* = 0.04). In subgroup analysis, these associations were limited to cranberry juice (SBP by 1.52 mmHg [95% CI − 2.97 to − 0.07; *P* = 0.05], DBP by 1.78 mmHg [95% CI − 3.43 to − 0.12, *P* = 0.04] and cherry juice (SBP by 3.11 mmHg [95% CI − 4.06 to − 2.15; *P* = 0.02]). Berries also significantly elevated sVCAM-1 levels by 14.57 ng/mL (85% CI 4.22 to 24.93; *P* = 0.02).

**Conclusion:**

These findings suggest that supplementing cranberry or cherry juice might contribute to an improvement in blood pressure. No other significant improvements were observed for other specified fruits. More research is warranted comparing different classes of fruit and exploring the importance of fruit processing on their cardiovascular-protective effects.

**Electronic supplementary material:**

The online version of this article (10.1007/s00394-020-02299-w) contains supplementary material, which is available to authorized users.

## Introduction

Current World Health Organization (WHO) recommendations for fruit intake combined with vegetable intake are a minimum 400 g/day [1]. A recent meta-analysis indicated that the intake of 800 g/day of fruit was associated with a 27% reductions in relative risk of CVD [2]. It is well recognised that cardiovascular health can be affected by several dietary factors [3]. Epidemiological evidence has established strong inverse associations between flavonoid-rich fruit (e.g. strawberries, grapefruit) and coronary heart disease (CHD) mortality in CVD-free postmenopausal women after multivariate adjustment [4]. Endothelial function is a primary indicator of cardiovascular health, a damaged endothelium will cause disruption of vascular hemostasis and further lead to endothelial dysfunction, which is the manifestation underlying atherosclerosis, hypertension, and other CVDs [5, 6]. Intervention studies also provide evidence supporting the consumption of a range of fruit and fruit juice to reduce cardiovascular dysfunction risk factors. For example, consumption of fruit containing relatively high levels of anthocyanins and procyanidins, such as berries, has been shown to improve CVD risk factors, namely endothelial dysfunction, dyslipidaemia, platelet aggregation, and hypertension [7, 8], whereas flavanone-rich citrus, such as orange, were effective in improving hypercholesterolaemia [7]. The consumption of cherries was also suggested by interventions to promote cardiovascular health by preventing or decreasing lipid levels and inflammation [9]. One systematic review and epidemiological evidence have also revealed that the consumption of fruit juice including citrus, berries and cherry juice may benefit vascular health by affecting risk markers such as blood pressure and lipid profiles [10, 11].

Fruit juice and powder may be effective methods to increase overall fruit consumption, which may explain the emerging intervention studies investigating health benefits with fruit powder and juice supplementations. With regard to nutritional value, freeze-dried fruit powder that is devoid of water retains concentrated bio-accessible antioxidants, fibre and other components [10]. Research has suggested that the juicing process can lead to a lower content of fibre and certain bioactives such as polyphenols, vitamins, and minerals [12, 13], while other research suggests that processing can increase the bioavailability of carotenoids, such as lycopene [14]. A recent single-dose bioavailability study showed only minor differences between whole blueberry fruit and blueberry juice [15]; indeed a systematic review demonstrated that the intake of fruit and vegetable juice offered similar cardiovascular health benefits to the intake of whole fruit and vegetables [16].

Other systematic reviews have assessed the effect of fruit and vegetable intake on endothelial function or the effect of specific fruit juice intake on CVD risk factors [17, 18]. However, to the best of our knowledge, the effects of the fruit-delivery method (type and processed form) in relation to CVD risk factors including endothelial (dys)function, lipid profile (i.e. total cholesterol) and inflammatory biomarkers (i.e. C-reactive protein/CRP) has not been appraised. A review of this type is important to clarify the evidence base for the type and form of fruit that is most cardiovascular-protective. Therefore, the aim of this study was to systematically review and meta-analyse available human intervention studies to evaluate the potential effect of consumption of whole, freeze-dried, powdered, and juiced forms of fruit, and specifically berry, citrus and cherry fruit, on CVD risk factors in randomised controlled trials (RCTs) in line with the PICOS (population, intervention, comparator, outcome, study design) framework (Supplemental Table 1).

## Methods

### Study eligibility

We searched for studies assessing the effect of specific fruit supplementations on CVD risk factors including terms of “fruit”, “CVD risk factors”, “endothelial function”, “BP”, “lipid”, “inflammatory biomarker”. The following specific inclusion criteria were applied: (1) study design: RCTs; (2) subjects: adult subjects ≥ 18 years of age; (3) interventions: intervention RCTs providing or promoting berry, or cherry or citrus fruit or their juice or freeze-dried, or powdered fruit consumption; (4) intervention length: at least 1 week; (5) control: control groups without components of citrus fruit, cherry, or berries, likely placebo group; (6) outcomes: the primary outcomes were the whole body measurements: systolic and diastolic blood pressure (SBP and DBP) and the endothelial (dys)function assessed by flow-mediated dilation (FMD) and pulse wave velocity (PWV); the secondary outcomes were the blood biomarkers including circulating fatty acids triglycerides (TAGs) and total cholesterol (TC), low-density lipoprotein cholesterol (LDL-C) and high-density lipoprotein (HDL-C); inflammatory biomarkers such as high-sensitivity C-reactive protein (hsCRP), nitric oxide (NO), intercellular adhesion molecules (ICAMs) and vascular adhesion molecules (VCAMs) were also explored (described below); (7) only English-language and peer-reviewed articles were included. No restriction of publication year was applied.

### Data sources

The present systematic review and meta-analysis was conducted in accordance with Cochrane [19] and Centre for Reviews and Dissemination guidelines [20] and was reported according to PRISMA guidelines [21] (Supplemental Table 2). The protocol has been registered with PROSPERO, the International Prospective Register of Systematic Reviews (Registration number CRD42018091896). Such protocol includes the investigation of the impact of these fruits on cognitive function, however this analysis will be reported elsewhere. Two researchers (YW, JLG) assessed articles independently for inclusion eligibility. The searches using PubMed, Web of Science, Scopus and psycARTICLES were conducted from inception until January 2020. No restriction of publication year was applied and the search result covered studies published between 1960 and 2020.

The search of the investigated themes in this review was undertaken using terms as following: (1) fruit; (2) citrus; (3) orange; (4) berry; (5) berries; (6) grape; (7) blueberry; (8) blueberries; (9) blackberry; (10) blackberries; (11) raspberry; (12) raspberries; (13) cranberry; (14) cranberries; (15) cherry; (16) cherries; (17) “endothelial function”; (18) “vascular function”; (19) “vascular risk factors”; (20) hypertension; (21) “blood pressure”; (22) BP; (23) “pulse wave velocity”; (24) PWV; (25) “flow-mediated dilation”; (26) FMD; (27) lipid; (28) cholesterol; (29) LDL; (30) HDL; (31) triglyceride; (32) biomarkers; (33) inflammatory; (34) “Nitric Oxide”; (35) NO; (36) ICAM; (37) VCAM; (38) hsCRP; (39) trial; (40) intervention. Search strategy was supplied (Supplemental Table 3).

### Study selection

YW and JLG selected articles independently for eligibility. Articles were moved to the next screening phase or discarded when full disagreement was reached. JKL served as an arbitrator if any disagreements that were not resolved. No disagreements occurred during the selection phase. All records were exported to EndNote X8 reference management software. The selection of eligible studies was based on two steps. Firstly, the title and abstract of each study were screened for relevance; full texts were then reviewed for those without certainty for inclusion. Reference lists of included papers and relevant systematic reviews were also screened by hand-searching for additional articles.

### Data abstraction

Data were extracted by YW and JLG independent of each other, their selections for accuracy were reviewed in meeting. Corresponding authors were contacted via e-mail to request information if there were missing data or for clarification. Data from endpoints and the baseline were obtained. A pre-defined data extraction form in Microsoft Excel 2016 was used to input studies data, which includes information on (1) author and published year; (2) study design; (3) population characteristics (ethnicity, mean age, sex, mean body mass index (BMI), health status and sample size at baseline); (4) treatment details (intervention type, length, dosage and frequency); (5) control group settings; (6) retention rate; (7) measured outcomes for both experimental group and placebo group at baseline and the longest post-intervention time point to avoid the bias of selectively choosing data.

### Risk of bias assessments

Study quality for RCTs was assessed by Jadad Score (0–5), which takes into account whether a trial was randomised and blinded with appropriate procedure, and whether dropouts were well recorded; a score ≥ 3 indicates a high-quality trial [22].

Publication bias was assessed by Funnel plot and Egger’s test, ‘trim and fill’ method was implemented to identify and correct for funnel plot asymmetry arising from publication bias [23] (Supplemental Fig. 1).

### Data synthesis

R studio version 3.5.2 [24] and the package “meta” [25] were used to pool and meta-analyse data from collected studies. Subgroup analysis with at least 10 studies supplementing berries was implemented to estimate separate effects of different types of berries and the heterogeneity for each berry intervention subgroup. There were 3 studies supplementing grapefruit juice and cherry juice as concentrate instead of 100% juice, which could cause variations to the juice quality and bioavailability [26–28]. For example, anthocyanins are better preserved in purees (57%) than in clarified juice (31%) when comparing different forms of processed blackberries [29]. Sensitivity analysis of juice quality was carried out to investigate the effect of juice on the meta-analysis results.

All pooled results were presented as weighted mean difference with 2-sided *p* values. 95% confidence intervals (CIs) and prediction intervals were both presented in the results. The FMD value was expressed in percentage unit and the PWV value was expressed in m/s; the conversion of cm/s to m/s for PWV value was applied when necessary for pooled mean differences in meta-analysis. For blood lipids, the conversion factor 1 mmol/L = 38.67 mg/dL was used for total, HDL, and LDL cholesterol level and 1 mmol/L = 88.57 mg/dL for triglycerides level [30] where applicable.

The Hartung–Knapp–Sidik–Jonkman method for random-effects meta-analysis [31] was applied. Heterogeneity was estimated by Cochrane *Q* statistics and the consistency of study results was assessed by *I*^2^ statistics as an extension of Cochrane *Q* statistics and an *I*^2^ > 50% is considered for a high heterogeneity level [32]. The effect sizes based on the weighted mean difference (WMD) between treatment groups were used when measurement units of assessed outcomes were comparable across studies. The standardised mean difference (SMD) was used when studies have used different measurement units and the conversion had failed.

## Results

### Literature search

**Fig. 1:**
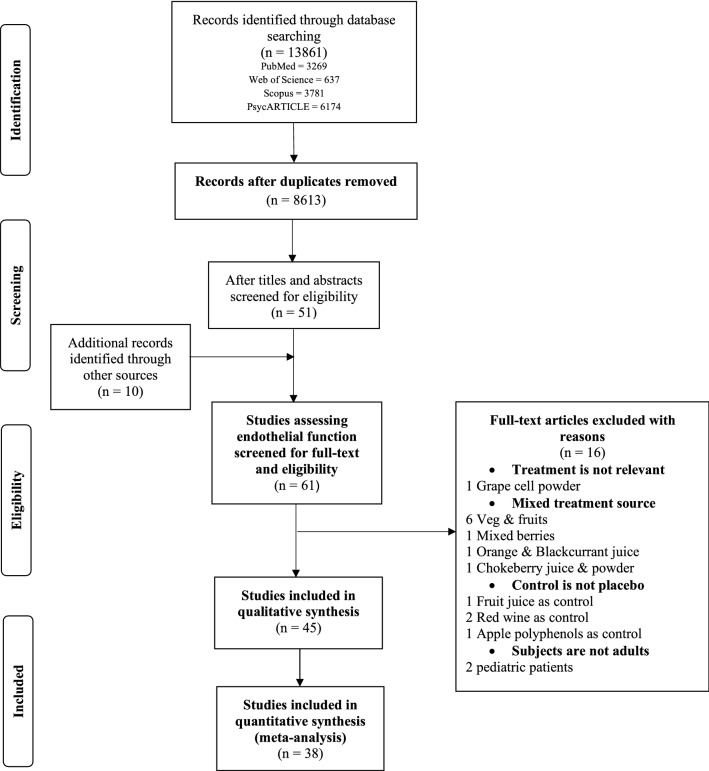
Flow diagram of study selection for the review according to PRISMA guidelines

In accordance with PRISMA guidelines [21], Fig. 1 describes the selection process of included studies. The initial search produced 13,861 articles from the four databases, this record was reduced to 8613 articles after duplicates were removed. After screening of the titles and abstracts for eligibility, 51 articles were included and 10 additional articles were added from manual search through reference lists of initially identified articles. The final selection identified 61 trials assessing CVD risk factors, where 16 articles were further excluded after checking full-text eligibility (Supplemental Table 4). Finally 45 trials were included in this review, 38 trials from these were included in the meta-analysis.

### Study characteristics

Forty-five studies were included in this systematic review, of which 18 were crossover randomised controlled trials (RCT) [27, 33–49], and 27 were parallel RCTs [26, 28, 50–74] (see Table 3). The sample size of both experimental and control group in the interventions ranged from 5 to 63. The total sample size for the intervention group was 1130; the total sample size for the control group was 1109. Participants’ characteristics at baseline also vary across studies; most trials recruited healthy subjects (*n* = 13), while there were 7 studies with participants manifesting increased CVD risks (deteriorated lipid profiles and hypertension) and 3 with diagnosed CVD/CHD; 18 with metabolic syndrome (inclusive of overweight); 1 with mild‑to‑moderate dementia, 1 with chronic obstructive pulmonary disease, 1 with type 2 diabetes and 1 with end-stage renal disease (Table 3).

Results from 32 studies (71% of the interventions) that supplemented fruit juice are shown in Table 1, while studies supplementing whole fruit and fruit in other freeze-dried forms (13 studies), are presented in Table 2. Study effects are represented with greyscales in corresponding with reported positive, negative effects and no effect compared to either control or baseline data. Treatments were all delivered in arms of experimental and control groups. The mean chronic treatment duration was 57 days with a standard deviation (SD) of 43 days (ranged from 7 days to 180 days).

**Table 1:**
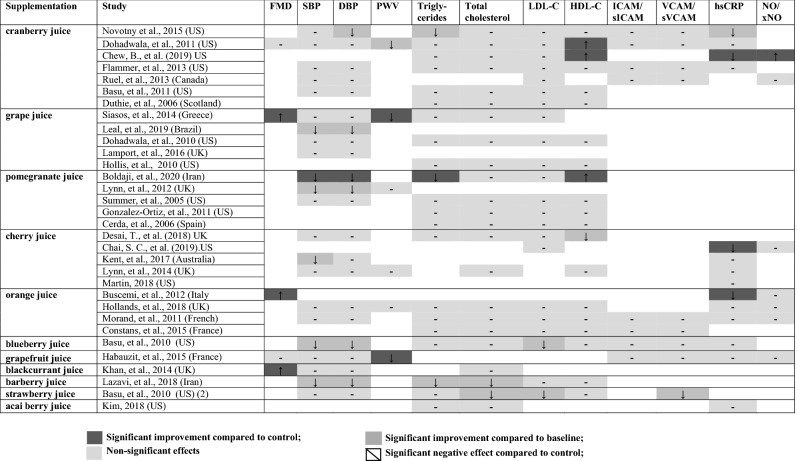
Qualitative summarisation for fruit juice interventions

**Table 2:**
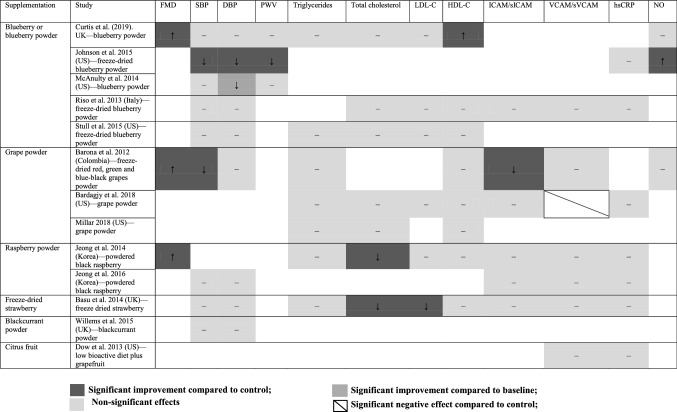
Qualitative summarisation for whole fruit or freeze-dried or powdered fruit interventions

Among the fruit juice category, most studies evaluated the effect of cranberry juice, grape juice, pomegranate juice, cherry juice, orange juice (*n* = 7, 5, 5, 5, 4, respectively). The mean dosage applied for these types of juices was 480 mL, 353 mL, 238 mL, 173.6 mL and 425 mL, respectively. The remaining interventions included blueberry juice (*n* = 1), grapefruit juice (*n* = 1), barberry juice (*n* = 1), blackcurrant juice (*n* = 1), strawberry juice (*n* = 1) and acai berry juice (*n* = 1) (see Table 1). In Table 2, four trials supplemented freeze-dried blueberry powder. Portion conversion of powder to whole fruit was provided in each study; typically the mean dosage of blueberry powder supplementations was 32.75 g (equivalent to approximately 1.5 cups of fresh blueberries). Three trials supplemented freeze-dried grape powder. The mean dosage of grape powder supplemented was 55.33 g, which is equivalent to approximately 2.5 cups of fresh grapes. The remaining 5 studies supplemented other berries (powdered raspberry, powdered blackcurrant, freeze-dried strawberry) and citrus fruit (1.5 portion of grapefruit following a low bioactive diet).

### Study quality

The average retention rate for all included trials was 92.64%, of which 30 out of 45 RCTs obtained no less than 3 points of total Jadad score (see Table 3). Trials generally provided adequate description of methods and procedures, although only 40% of RCTs implemented true randomisation with an adequate description of methods (i.e. computerised statistical randomisation) and 33.33% of RCTs reported implementing blinding processes, where the placebo were colour and taste matched to mask treatments, and the received treatment was not revealed until the statistical analysis was completed for double blinding. However, there was no report assessing participants’ blinding for instance by guessing the treatment they received (Supplemental Table 5).

**Table 3:** Summary of fruit interventions

(Author/year) country of origin	Study design	Ethnicity (%)	Mean or range age (years)	Sex (%male)	Mean BMI (kg/m^2^)	Participants healthy status	Baseline sample size (total, intervention, control)	Intervention type	Intervention length (Hours(h), Days(d) or Weeks (wks))	Dose and frequency of consumption	Control group	Retention rate	Outcomes	Total Jadad score (0–5)
Bardagjy et al. (2018) US	Randomized, placebo controlled, double-blinded crossover trial	NA	48.6 ± 15.4	20%	37.0 ± 9.9	Obese but otherwise healthy adults	23– > 20	60 g equals 2.2 cups or 330 g of grapes	4 wks	GP (60 g) was equivalent to 330 g or 2.2 cups of fresh grapes and contained 297 mg total polyphenols (as gallic acid equivalents)	PBO was matched to GP in calories, macronutrients, taste, and appearance but provided zero polyphenols/serving	87%	SBP, DBP, TC, HDL, LDL, TG, CRP, ET-1, IL-6: oxLDL, sICAM, sVCAM, TNFc	3
Barona et al. (2012) Colombia	Double-blind cross-over RCT	N/A	51.3 ± 9.6	N/A	N/A	Metabolic syndrome	25	grape powder	30d	46 g/d = 2 serving of fresh grapes	Placebo	96%	SBP, DBP, FMD, TG, HDL, Glucose, BMI, Nox, sICAM-1, sVCAM-1, E-selectin	2
Boldaji et al. (2019) Iran	Crossover RCT	Pomegranate juice	47.8 ± 13.3	61%	23.9 ± 4.8	ESRD patients on dialysis treatment, dialysis 3 times a week for at least 3 months,	40:38	100 mL PJ	8 wks	100 mL PJ three times a week after their dialysis session	No intervention as control	97.6%	SBP, DBP, TC, HDL, LDL, TG, IL-6	2
Buscemi et al. (2012) Italy	Single-blinded crossover RCT	N/A	48 ± 13	53	32.1 ± 4.9	subjects with increased CVD risk	19	red orange juice	7d	500 mL/d	Placebo drink (12 healthy non-diabetic subjects acting as control group)	91%	FMD, GTN (glyceryl-trinitrate), hs-CRP, IL-6, TNF-α, NO, PCs (protein carbonyl)	2
Constans et al. (2015) France	Cross-over, single-blinded RCT	N/A	53.8 ± 2	100	26 ± 1	Mild hypercholesterolemic men (LDL-C between 130 and 190 mgL)	25	Blond orange juice	4 wks	200 mL (3×/d)	Control beverage	96%	Glucose, TC, LDL, HDL,TC/HDL, TG, ApoA-1, ApoB, Lpa, hsCRP, Brachial FMD, sICAM-1, sVCAM-1, sE-selectin	3
Desai et al. (2018) UK	Single-blinded crossover RCT	Montmorency tart cherry juice (MTCJ)	30 ± 10 years	7/11	BMI 24.43 ± 3.23	Healthy	11	30 mL MTCJ	20 days	30 mL	Placebo	100%	SBP, DBP, TC, LDL, TG, IL-7	3
Dohadwala et al. (2010) US	Crossover RCT	42% black	43 ± 12	69	28 ± 3.8	Stage 1 hypertension	63	Concord grape juice	8 wks	490 mL (965 mg/d)	Placebo drink	77%	SBP, DBP, TG TC, LDL, HDL	3
Dohadwala et al. (2011) US	Crossover double-blind RCT	Black 45.5%	62 ± 10	68	29.5 ± 4.5	Coronary heart disease	44	Double-strength cranberry juice	4 wks	480 mL/d	Calorie, taste, and appearance-matched placebo beverage containing no polyphenols	91%	SBP, DBP, FMD, Baseline diameter, Dilation to nitroglycerin, Baseline flow, FMD, Hyperemic flow, InPAT ratio, Cartoid-radial PWV, Cartoid-femoral PWV	4
Habauzit et al. (2015) France	Crossover double-blind RCT	Caucasian	58 ± 4	0	25.7 ± 2.3	Postmenopausal woman	52	Concentrate blond grapefruit juice	6 months	340 mL/d (2 × 170)	Isocaloric Control drink	92%	SBP, DBP, Pulse pressure, FMD dilation, Baseline brachial diameter, PAT ratio, Pulse pressure, NO, Endothelin 1	4
Holland et al. (2018) UK	Open label crossover RCT	Blood orange juice	52.2 ± 13.6	Male 20/41	29.0 5.1	Healthy	45 (41)	500 mL blood OJ providing 50 mg anthocyanins/d	28 days	2 × 250– > 500 mL	500 mL blonde OJ without anthocyanins	91%	SBP, DBP, ba PWV, cf PWV, NO, CRP, TG, HDL-C, LDL-C	2
Lamport et al. (2016) UK	Double-blind, crossover RCT	N/A	43.2 ± 0.6	0	24.6 ± 0.5	Healthy	25(19)	Concord grape juice	6 wks, 12 wks	355 mL/d	Energy-, taste-, and appearance-matched placebo	77%	SBP, DBP	4
Martin (2018) US	Crossover RCT	N/A	38.1 ± 12.5 years	###	32.2 ± 4.6:32.2 ± 4.8	Overweight and obese subjects	13-- > 10	240 mL tart cherry juice	4 weeks	240 mL/d	Placebo juice	77%	IL-6, IL-10, TNF-, MCP-1, hsCRP	4
Millar (2018) US	Double-blind crossover RCT	N/A	53.5 ± 10.1	###	33.0 ± 4.77 kg/m^2^	Metabolic syndrome	20	Grape powder (contributing 195 mg total polyphenols)	4 weeks	60 g grape powder per day	Placebo powder	100%	Total cholesterol, HDL-C and HDL particles, TG	3
Morand et al. (2011) French	Crossover RCT	N/A	56 ± 1	100	27.4 ± 0.3	Overweight healthy	24	Orange juice	4 wks	500 mL/d	Control drink + placebo capsules (starch)	100%	SBP, DBP, Pulse pressure, Glucose, Insulin, Triglycerides, Total cholesterol, LDL, HDL, CRP, IL-6, vWF, sICAM-1, sVCAM-1, NOx	5
Riso et al. (2013) Italy	Crossover RCT	N/A	47.8 ± 9.7	100	47.8 ± 9.7	CVD risk factors	18	Freeze-dried wild blueberry powder 25 g	6 wks	250 mL/d	Placebo drink consisted of 250 mL water, 7.5 g fructose, 7 g glucose, 0.5 g citric acid and 0.03 g blueberry flavor	89%	RHI, FRHI, AI, AI@75, Diastolic pressure, SBP, Total NO, sVCAM-1	2
Ruel et al. (2013) Canada	Double-blind crossover RCT	N/A	45 ± 10	100	28.3 ± 2.4	Overweight	35	27% cranberry juice	4 wks	500 mL/d	Placebo juice	100%	Heart rate, Systolic BP, Diastolic BP, MAP, Resting AIx, Δ AIx salbutamol, Δ AIx GTN, Global endothelial function, NOx, Uric acid, Oxidized LDL, sICAM-1, sVCAM-1, sE-selectin	3
Siasos et al. (2014) Greece	Double-blind crossover RCT	N/A	26.34 ± 4.93	38	23.21 ± 4.10	Healthy	26	100% concord grape juice	7 d, 14 d	7 cc/kg/d	The grapefruit placebo juice matched the flavor, color, calorie, and sugar profile of the CGJ but did not contain any polyphenols	100%	FMD, PWV/carotid-femoral, Total cholesterol, LDL-C, TG, Serum glucose, SBP, DBP	4
Willems et al. (2015) UK	Double blind, randomized, placebo-controlled, and cross-over	N/A	38 ± 8	62	23 ± 2	Healthy	13	Blackcurrant powder	7 d	6 g/day (138.6 mg anthocyanins)	Blackcurrant juice 3–4 mg anthocyanins per dose	77%	SBP, DBP, mean arterial BP, heart rate, Stroke volume, cardiac output, peripheral resistance	2
Basu et al. (2014) UK	RCT	N/A	49 ± 10 y	9.1	36 ± 6 5	Obese adults with elevated serum lipids	60 (15:15:15:15)	High-dose freeze dried strawberry (10% weight of fresh strawberries) and low-dose freeze-dried strawberry	12 wks	50 g/d for high dose; 25 g/d for low dose	High-dose calorie- and fiber-matched control 44 g/d; low-dose calorie- and fiber-matched control 24 g/d	100%	BMI, SBP, DBP, glucose, insulin, Total cholesterol, LDL-C, HDL-C, LDL:HDL, VLDL-C, TGs, hs-CRP, sVCAM-1, sICAM-1	2
Basu et al. (2010) US	RCT	N/A	47.0 ± 3.0	7.4	37.5 ± 2.15	Metabolic syndrome	30 (15:12)	Freeze-dried strawberry beverage (50 g freeze-dried strawberries ∼ 3 cups fresh strawberries	8 wks	3 cups/d	4 cups of water/d	90%	BMI, SBP, DBP, glucose, Total cholesterol, HDL-C, LDL-C, VLDL-C, TGs, ICAM-1, VCAM-1	2
Basu et al. (2011) US	RCT	N/A	52.0 ± 8.0	0	40.0 ± 7.7	Metabolic syndrome	31 (15:16)	Cranberry juice	8 wks	240 mL/458 mg/d	Placebo drink	97%	SBP, DBP, Total cholesterol,H DL-C, LDL-C, TGs	3
Basu et al. (2010) US	Single-blinded parallel RCT	N/A	50.0 ± 3.0 SE	8.3	37.8 ± 2.3	Metabolic syndrome	66 (25:23)	480 mL freeze-dried blueberry beverage (50 g freeze-dried blueberries)	8 wks	50 g freeze-dried bb beverage	480 mL water and vanilla extract	73%:72%	SBP, DBP, TG, Total, LDL-, HDL-, oxLDL- cholesterol	2
Cerda (2006a) Spain	RCT	N/A	60 ± 10.9	N/A	31.4 ± 4.8	Chronic obstructive pulmonary disease	30 (15:15)	400 mL pomegranate juice (2660 mg/d)	5 wks	400 mL/d	Placebo drink	100%	Total cholesterol, HDL-C, LDL-C, TGs	3
Curtis et al. (2019) UK	Double-blind, parallel RCT	NA	63 ± 7	###	31.2 ± 3.0	Metabolic syndrome	144– > 115(37:39:39)	26 g equivalent to 1 cup (150 g) and 1/2 cup (75 g) milled blueberries powder	6 months	Equivalent 150 g BB	Dextrose, maltodextrin, and fructose, which were produced as a purple powder, with blueberry aromatics generated from natural (nonanthocyanin) and artificial color and flavorings	80%	SBP, DBP, TC, HDL, LDL, TG	3
Chew et al. (2019) US	Randomized, double-blind, placebo-controlled, parallel design trial	NA	43.1 ± 1.1	###	30.8 ± 0.4 k	Non-smoking overweight	79– > 78(40:38)	450 mL cranberry extract beverage (CEB)	8 wks	450 mL	The placebo beverage was designed to look, smell, and taste such as the CEB, but did not contain cranberry constituents.	98%	CRP, IL-6, IL-10, IL-23, TNF-α; IFN-γ	3
Chai et al. (2019) US	Parallel RCT	Tart cherry juice	28.5 ± 3.7:27.3 ± 4.2	40%:53%	70.0 ± 3.7:(69.5 ± 3.9	Older adults	37– > (20:17)		12 weeks	68 mL of Montmorency tart cherry concentrate was diluted with 412 mL of water	Control drink was prepared by mixing unsweetened black cherry flavored Kool-Aid (Kraft Foods, Chicago, IL, USA) with water	100%	TNF-α, CRP, ET-1, NO, OxLDL	3
Duthie (2006b) Scotland	RCT	N/A	18-40 y	0	N/A	Healthy	20 (11:9)	Cranberry juice	2 wks	750 mL (852 mg/d)	Placebo drink (6.72 mg/d)	100%	Total cholesterol, HDL-C, LDL-C, TGs	2
Dow et al. (2013) US	RCT	Non-Hispanic white race (62.3)	41.8 ± 10.7	30	32.1 ± 4.1	Obese or with additional MetS (42%)	74 (37:32)	Low bioactive diet plus half of a fresh Rio red grapefruit × 3 times	6 wks	Low bioactive diet plus 1.5 grapefruit/d	A low bioactive diet devoid of citrus	93%	sVCAM-1, hsCRP	1
Flammer et al. (2013) US	Double blind RCT	N/A	49.5 ± 16.2	45	27.7 ± 5.9:27.2 ± 5.5	Peripheral endothelial dysfunction and CVD risk factors	84 (32:37)	Cranberry juice ((double-strength Ocean Spray^®^ light cranberry juice cocktail (54% cranberry juice))	4 months	2*230 mL/d	Placebo juice beverage, an isocaloric formulation mimicking the flavor and color of the cranberry beverage	82%	RHI, SBP, DBP, pulse pressure, heart rate, AI augmentation via EndoPAT, hsCRP, VCAM, ICAM, I1-6, TNF-alpha, oxLDL, Cholesterol, HDL, TG	3
Gonzalez-Ortiz (2011b) US	RCT	N/A	25–55 y	N/A	30.0–39.9	Obesity	20 (10:10)	Pomegranate juice	1 month	120 mL	Placebo drink	100%	Total cholesterol, HDL-C, LDL-C, TGs	3
Hollis (2010a) US	RCT	N/A	18–55	N/A	25.0–29.9	Overweight	51 (25:26)	Concord grape juice	12 weeks	480 mL (933.6 mg/d)	Placebo drink	100%	Total cholesterol, HDL-C, LDL-C, TGs	3
Jeong et al. (2014) Korea	Double-blind parallel group RCT	N/A	58.0 ± 9.2:60.1 ± 9.5	47	26.3 ± 4.3:25.1 ± 4.0	Metabolic syndrome	77(39:38)	Powdered black raspberry	12 wks	750 mg/d (4 capsules)	Placebo group-cellulose, isomalto, and corn powder.	92%	Resting brachial artery diameter, reactive hyperemia brachial artery diameter, IL-6, NF-a, C-reactive protein, Adiponectin, sICAM-1, sVCAM	3
Jeong et al. (2016) Korea	Double-blind RCT	N/A	56.4 ± 9.2:60.7 ± 10.4	N/A	25.9 ± 4.6:24.7 ± 3.9	Metabolic syndrome	51(26:25)	Black raspberry powder	12 wks	750 mg/d (4 capsules)	Placebo	100%	SBP, DBP, heart rate, radial augmentation	3
Johnson et al. (2015) US	Double-blind parallel group RCT	N/A	59.7 ± 4.58:57.3 ± 4.77	1	30.1 ± 5.94:32.7 ± 6.80	Pre- and stage 1-hypertension	49 (20:20)	Freeze-dried blueberry powder	4 wks, 8 wks	22 g/d	22 g macronutrient-matched control powder consisted of maltodextrin, fructose, artificial and natural blueberry flavoring, artificial purple and red color, citric acid, and silica dioxide	83%	SBP, DBP, Mean arterial pressure, Carotid-femoral pulse wave velocity, Brachial-ankle pulse wave velocity, Heart rate	4
Kent et al. (2017) Australia	parallel groups RCT	N/A	78.9 ± 5.2:80.6 ± 6.6	51	25.7 ± 3.4:26.6 ± 3.5	Mild-to-moderate dementia	49 (24:25)	Cherry juice	12 wks	200 mL/d	Flavonoids-devoid apple juice	86%	Letter verbal fluency (executive function), SBP, DBP, heart rate, IL 6, hsCRP	4
Khan et al. (2014) UK	Parallel groups RCT	N/A	51 ± 11:51 ± 8	67	29.2 ± 6.9:28.9 ± 6.5	Healthy	66 (21:22:21)	High blackcurrant juice drink; low blackcurrant juice drink	6 wks	250 mL	Flavored water	97%	SBP, DBP, FMD, GTN-mediated vasodilation	3
Kim (2018) US	Double-blinded, placebo-controlled RCT	N/A	46.6 (11.5):42.0 (14.4)	31.6%:27.8%	33.5 ± 6.7	Metabolic syndrome	43– > 37(19:18)	açaí beverage (containing 1139 mg L − 1 gallic acid equivalents of total polyphenolics)	12 weeks	325 mL/d	325 mL placebo beverage	86%	Total cholesterol, TGs, hs-CRP, IL-6, TNF-a	2
Lynn et al. (2012) UK	Parallel groups, single blind RCT	N/A	39 ± 1.24 vs 36.1 ± 0.92	33	24.99 ± 1.26 vs 24.99 ± 1.06	Healthy	51 (24:24)	Pomegranate juice	4 wks	330 mL/day	lemonade drink-devoid of bioactive plant compounds, antioxidants or vitamins, and contained only a trace amount of sodium, similar energy and carbohydrate	100%	PWV, SBP, DBP, MAP, Heart rate	3
Lynn et al. (2014) UK	Parallel open-label RCT	N/A	38.3 ± 6.16 vs 37.2 ± 5.78	38	24.6 ± 3.63 vs 23.5 ± 3	Healthy	47 (25:21)	Cherry juice trate (30 mL diluted with 220 mL of water; Cherry Active^®^)	6 wks	250 mL/d	Lemonade drink	98%	PWV, hsCRP, SBP, DBP, Total cholesterol, HDL-C	2
Lazavi et al. (2018) Iran	Paralleled RCT	Barberry juice (BJ)	56.86 ± 8.47	33.3%:38%	29.22 ± 3.98:27.78 ± 3.45	Patients with type 2 diabetes (T2DM)	46 (23:23)	200 mL/d PJ	8 wks	200 mL	No intervention	100%	SBP, DBP, TC, HDL, LDL, TG, ApoB, ApoA	5
Leal et al. (2019) Brazil	Paralleled RCT	Grape juice	64.9 ± 4.0:72.9 ± 5.6	38.5%	24.7 ± 1.0:26.6 ± 1.1	22/31 hypertensive elderly	10:10	200 mL of GJ	12 wks	200 mL	No intervention	91%	SBP, DBP	1
McAnulty et al. (2014) US	Parallel groups RCT	N/A	46.15 + 11.92 vs 39.92 + 13.38	N/A	27.8 ± 5.46 vs 24.23 ± 3.44	Sedentary males and females	25 (13:12)	Blueberry powder--equivalent to 250 g rehydrated berries	6 wks	38 g/d	placebo powder contained a blend of maltodextrin, fructose, BB flavoring, coloring, citric acid, and a flow agent (silica)	100%	SBP, DBP, Aix (Augmentation Index), ASP (aortic systolic pressure), cPWV	2
Novotny et al. (2015) US	Parallel groups RCT	N/A	49.8 ± 11.1 vs 50.0 ± 11.6	48	27.8 ± 3.8 vs 28.9 ± 4.5	Healthy	60 (30:30)	CRANBERRY juice	8 wks	480 mL/d	Flavor/color/energy-matched placebo beverage	93%	Total cholesterol, LDL cholesterol, HDL cholesterol, TGs, apo A-I, apo A-II, apoB, sICAM, sVCAM, Diastolic BP, Systolic BP, CRP	4
Sumner et al. (2005) US	RCT	86.67% white	69 ± 11	89	28 ± 6	CHD and myocardial ischemia patients	45 (26:19)	Pomegranate juice	3 months	240 mL/d	Placebo drink	93%	SBP, DBP, Total cholesterol, HDL-C, LDL-C, TGs	4
Stull et al. (2015) US	parallel group double-blind RCT	52.27	55 ± 2:59 ± 2	36	35.2 ± 0.8 vs 36.0 ± 1.1	Metabolic syndrome	46(23:23)	Freeze-dried blueberry powder-- > 2 cups of fresh whole blueberries/consumed with 24-oz yogurt and skim milk-based smoothie	6 wks	45 g/d	Identical smoothie without the blueberry powder	87%	Glucose, Insulin, Triglycerides, Cholesterol, LDL, HDL, 24 h-SBP, 24 h-DBP, RHI	4

### Meta-analysis of CVD risk factors

**Fig. 2:**
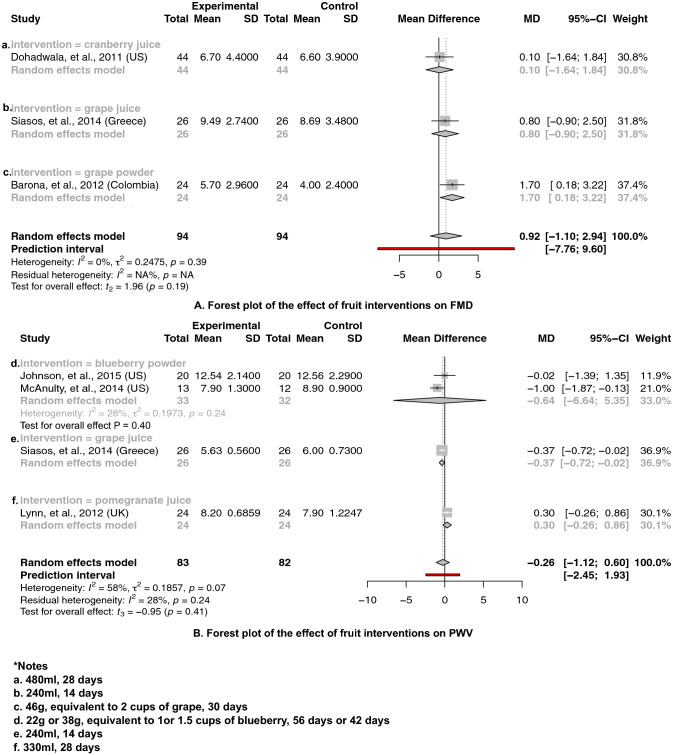
The effect of berry interventions including **a** cranberry juice, **b** grape juice and **c** grape powder assessing FMD and **d** blueberry powder, **e** grape juice and **f** pomegranate juice assessing PWV

**Fig. 3:**
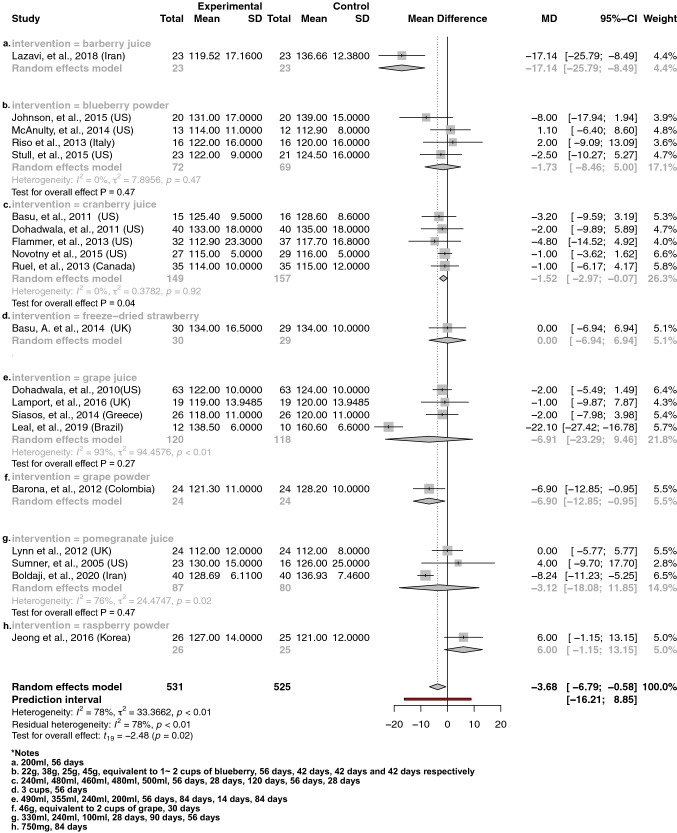
The effect of berry interventions including **a** barberry juice, **b** blueberry powder, **c** cranberry juice, **d** freeze-dried strawberry, **e** grape juice, **f** grape powder, **g** pomegranate juice and **h** raspberry powder assessing SBP

**Fig. 4:**
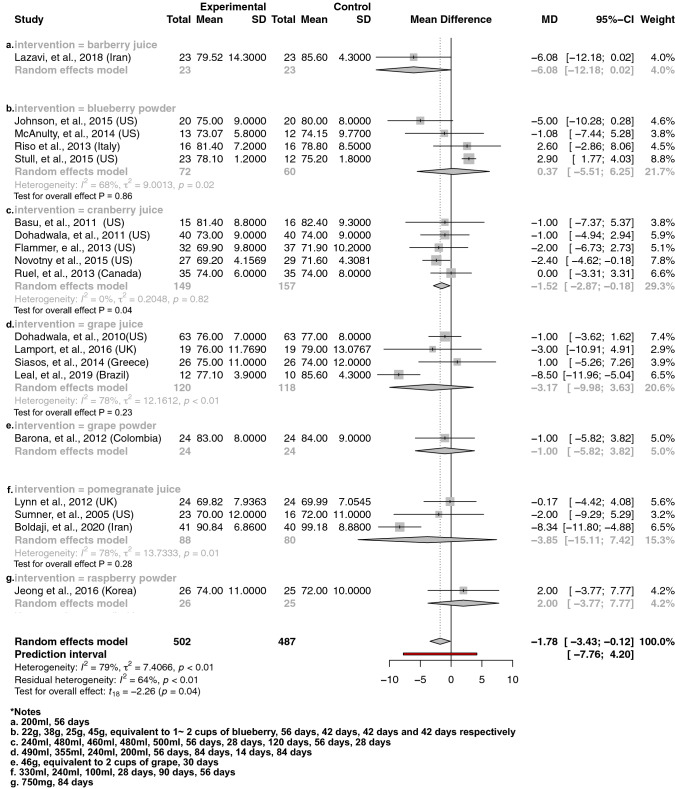
The effect of berry interventions including **a** barberry juice, **b** blueberry powder, **c** cranberry juice, **d** grape juice, **e** grape powder, **f** pomegranate juice and **g** raspberry powder assessing DBP

**Fig. 5:**
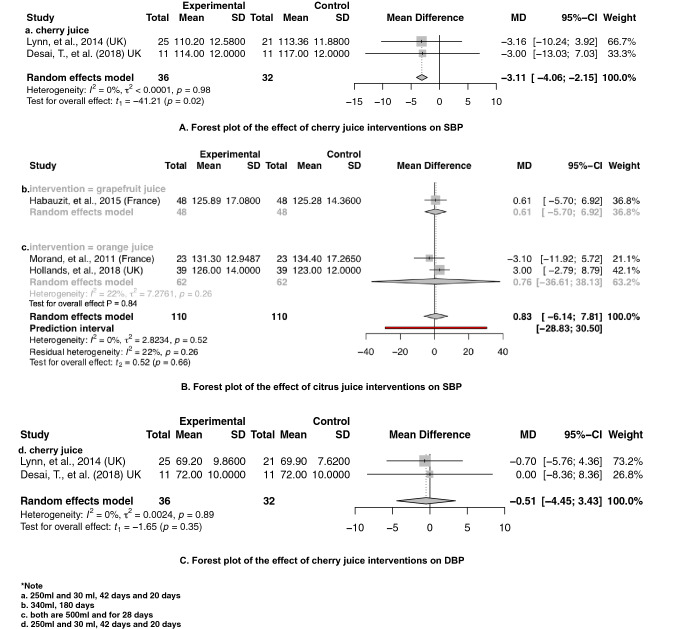
The effect of **a** cherry juice and **b** grapefruit juice, **c** orange juice interventions assessing SBP and **d** cherry juice interventions assessing DBP

Thirty-eight trials were included in the meta-analysis. The meta-analysis of 38 studies assessing FMD, PWV, SBP, DBP, levels of TAG, TC, HDL-C and LDL-C and levels of vascular inflammatory biomarkers ICAMs, VCAMs, hsCRP and NO are displayed in forest plots (Figs. 2, 3, 4, 5 and Supplemental Figs. 2–17). The interventions used in these studies supplemented: blueberry powder, grape juice and grape powder, cranberry juice, orange juice, whole grapefruit, pomegranate juice, raspberry powder, freeze-dried strawberry, acai berry juice and barberry juice. Among investigated outcomes, no significant improvements were shown to either FMD or PWV in the treatment group relative to the control group (Fig. 2). The *I*^2^ test suggested no heterogeneity for interventions assessing the effect on FMD (*I*^2^ = 0*%, P* = 0.39) and non-significant moderate heterogeneities for interventions assessing the effect on PWV (*I*^2^ = 58*%, P* = 0.07).

Our principal findings from a meta-analysis of interventions supplementing with berries (including 531 and 502 treatment participants) including barberry juice, blueberry powder, cranberry juice, freeze-dried strawberry, grape juice, grape powder, pomegranate juice and raspberry powder suggested significantly reduced SBP by 3.68 mmHg [95% CI − 6.79 to − 0.58; *P *= 0.02] (Fig. 3) and DBP by 1.78 mmHg [95% CI − 3.43 to − 0.12; *P *= 0.04] (Fig. 4), respectively. Subgroup analysis showed that specific interventions using cranberry juice, with mean dosage of 432 mL and length of 8 weeks, included 149 treatment participants and significantly decreased SBP and DBP by 1.52 mmHg (95% CI − 2.97 to − 0.07; *P* = 0.05) (Fig. 3) and 1.52 mmHg (95% CI − 2.87 to − 0.18, *P* = 0.04) (Fig. 4), respectively. Two cherry juice interventions including 36 treatment participants with dosage of 30 mL for 20 days and 330 mL for 6 weeks separately also led to a significant reduction in SBP by 3.11 mmHg (95% CI − 4.06 to − 2.15; *P* = 0.02) (Fig. 5). Berry group including blueberry juice, cranberry juice, grape powder, pomegranate juice and raspberry powder was also shown to significantly increase sVCAM-1 level by 14.57 ng/mL (95% CI 4.22 to 24.93, *P* = 0.02) in the treatment group relative to the control (Supplemental Fig. 12). The sensitivity analysis suggested no effect of grapefruit concentrate juice on the result of SBP and no effect of cherry concentrate juice on the results of SBP, DBP (Supplemental Table 6). The *I*^2^ test suggested significant substantial heterogeneities for berry group investigating the effects on SBP (*I*^2^ = 78%, *P* < 0.01) (Fig. 3) and DBP (*I*^2^ = 78%, *P* < 0.01) (Fig. 4). Funnel plots and the Egger’s test for the berry group showed an overall symmetric distribution of the interventions around the standard error for the investigated outcomes of SBP; asymmetric distributions were shown for the berry group investigating the effect on DBP, trim and fill method was further implemented to adjust for the publication bias (Supplemental Fig. 1).

There were no significant effects of other included intervention groups on other vascular and inflammatory markers: TAG (Supplemental Figs. 2, 3), TC (Supplemental Figs. 4, 5), LDL-C (Supplemental Figs. 6, 7), HDL-C (Supplemental Figs. 8, 9), ICAMs (Supplemental Figs. 10, 11), VCAMs (Supplemental Figs. 12, 13), NO (Supplemental Figs. 14, 15), or hsCRP (Supplemental Figs. 16, 17). The *I*^2^ test suggested significant substantial and moderate heterogeneities for berry group (*I*^2^ = 71%, *P* < 0.01) and cherry juice (*I*^2^ = 55%, *P* = 0.14) investigating the effects on TC, respectively (Supplemental Figs. 4, 5). There are significant moderate heterogeneity for berry group investigating the effects on HDL-C (*I*^2^ = 56%, *P* < 0.01) (Supplemental Fig. 8); non-significant moderate heterogeneities were shown for berry group investigating the effects on TAG (*I*^2^ = 36%, *P* = 0.08) (Supplemental Fig. 2), LDL (*I*^2^ = 37%, *P* = 0.08) (Supplemental Fig. 6). Funnel plots and the Egger’s test for the berry group showed an overall symmetric distribution of the interventions around the standard error for the investigated outcomes of TAG, TC, LDL-C (Egger’s tests *P* > 0.05) (Supplemental Fig. 1). Asymmetric distributions were shown for the berry group investigating the effect on TAG, trim and fill method was further implemented to adjust for the publication bias (Supplemental Fig. 1).

## Discussion

### Principal findings

We are continually reminded of the health benefits of consuming more fruit and one consumer-friendly strategy to increase fruit consumption is through juice [12]. Even though the juicing process can influence the nutritional value of fruit; a systematic review has demonstrated that the intake of fruit and vegetable juice offered similar health benefits to the intake of whole fruit and vegetables [16]. The results from our review support the beneficial effects of juice and have revealed the potential of berries, in juiced form, to play a beneficial role in the diet to maintain cardiovascular health. High dose of 432 mL cranberry juice and small studies of cherry juice using up to 330 mL showed improvements to blood pressure in our meta-analysis, whereas the National Health Service adult portion size recommendation for fruit juice is no more than 150 mL per day [75], thus a downsized portion according to daily recommendation should be studied in intervention studies.

These findings suggest that interventions with berries, especially using juiced cranberries or cherries, as the most active substitutes for whole fruit, may effectively reduce SBP and DBP. However, the current analyses do not support the notion that the consumption of fruit powders or other fruit juices will confer a cardiovascular function-protective benefit.

### Scientific analysis of findings

Our review showed that blueberry and grape in both juiced and freeze-dried forms have been frequently studied for their cardio and vascular protective effects, however, this quantitative analysis only supported an improvement on the outcomes by the consumption of cranberry juice and cherry juice.

A previous systematic review investigated the impact of fruit polyphenols on blood lipids (*n* = 17), platelet function (*n* = 9), BP (*n* = 9) and endothelium-dependent vasodilation (vascular function) (*n* = 7) and suggested that polyphenols from fruits such as pomegranate, purple grapes and berries are particularly effective at preventing hypertension compared to other CVD risk factors [7]. Berries in particular were shown to possess cardio-protective properties; the underlying mechanisms highlighted include inhibitory effects on inflammatory gene expression, oxidative stress, carbohydrate digestive enzymes and foam cell formation as well as increased effect on nitric oxide synthase following anthocyanins, the major polyphenol in berries [8].

A previous meta-analysis has grouped RCTs without separating the type of fruit, thus the magnitude of the effects of different fruit juice interventions were not compared. Their results supported the overall consumption of various fruit juices to significantly lower DBP by 2.07 mm Hg (95% CI − 3.75 to − 0.39; *P* = 0.02), whereas no improvement in SBP or lipid levels was obtained within 8 included RCTs [76]. In comparison with this previous report, the present report confirms the significant effects on DBP and reveals also significant effects on SBP.

In another meta-analysis of 95 prospective studies of fruit and vegetable intake, Aune et al. [2] found that fruit juice intake had little association with CVD and total cancer, while slight inverse associations were observed for CHD with RR (95% CI) 0.79 (0.63–0.98), stroke with RR (95% CI) 0.67 (0.60–0.76) and all-cause mortality with RR (95% CI) 0.87 (0.83–0.91) every 100 g/day increment, however, the very low number of studies (*n* = 2) makes these findings preliminary and more studies are needed before any firm conclusions can be drawn [2]. Furthermore, there is evidence showing that increasing the consumption of fruit juice by one serving per day was associated with a 7% greater incidence of type 2 diabetes (95% CI 0.8% to 14%) [77] and there is also greater risk of weight gain with higher consumption of fruit juice, probably because of the high sugar content and excess calories provided [78]. Fruit juice contains quantities of sugar classified as ‘free’ sugars like sucrose, compared with whole fruit in which the sugars are classified as intrinsic. Increased dietary fructose following sucrose intake is reported to increase de novo lipogenesis (DNL) levels and VLDL, which has been shown to increase the risk of developing non-alcoholic fatty liver disease (NAFLD) [79]. Therefore, cautious interpretations should be made when promoting fruit juice consumption as healthy options to increase fruit and vegetable intake.

Other epidemiological evidence has indicated an inverted association between fruit intake and CVD risk factors. Among 34,492 CVD-free postmenopausal women in the Iowa Women’s Health Study with 16 years of follow-up, a significantly reduced risk ratio of CVD mortality was associated with intake of at least once per week of apples and pears, oranges, grapefruit, blueberries, strawberries, grapes and raisins after adjustment for age and energy [4]. However, following adjustment for other confounding covariates, the significance was only retained for the intake of strawberries, apples and pears. In a further investigation of strawberry intake for its cross-sectional association with lipids and CRP profiles, only a borderline significance was reported for a reduced CRP levels [80]. Aune et al. [2] also reported an inverse association between high vs. low berry consumption and all-cause mortality in a meta-analysis, whereas no similar associations were observed for CHD and CVD [2].

Our review has also shown elevated sVCAM-1 level after the berries intervention, however, some authors have suggested that the magnitude of the increase in sVCAM-1 may not be clinically relevant, as the other vascular inflammatory markers did not change between the treatment and the control group after the interventions [41], which is also in line with the results of other inflammatory markers in our review. Aside from this, Bardagjy et al. [41] and Ruel et al. [44] reported significantly higher sVCAM-1 levels in the treatment group at the baseline compared to the control, which may have contributed to the elevated sVCAM level after the interventions in the berries-treated group. Although the consumption of a range of berries have been linked with improved cardiovascular health, considering the results from our review and previous evidence, current evidence is insufficient and inconsistent to substantiate the consumption of specific berries or other fruit as a cardiovascular-protective dietary strategy.

### Implications for health and future research

Among our results, SBP improved significantly by over 3 mmHg after interventions with specific berries and cherry juice, which may likely have practical implications as blood pressure is an important indicator not just for endothelial function, but also for CVD mortality risk [81]. A report from the Joint National Committee and several meta-analyses have estimated that lowering SBP by 5 mmHg or more could decrease stroke risk by 13% [82], CVD risk by 3% to 38% [83], deaths from stroke by 14%, deaths from heart diseases by 9% and overall mortality by 7% [84].

However, we only analysed two cherry juice studies with relatively small sample sizes in this review and no other risk factors were improved by this intervention. It would be helpful to have more studies on this topic in order to inform policymakers in nutrition. Future studies on supplementing berries (i.e. cranberries, blueberries, grapes) with a sufficient sample size are warranted, as these appear to have the biggest potential to improve endothelial function and cardiovascular function. Further studies on this topic incorporating effect sizes with interpretation from CVD risk reduction are also required.

### Strengths and limitations

To our knowledge, this is the first systematic review and meta-analysis to compare the impact of fruit in various delivery forms, on cardiovascular health. We also used the newly developed Hartung–Knapp–Sidik–Jonkman method for random-effects model in meta-analysis in addition to a comprehensive search of the literature in the topic. There are limitations to our review, however. As explored by the subgroup analysis, the significant moderate-to-substantial heterogeneity among the berry group majorly contributed to grape juice and pomegranate juice studies (Figs. 3, 4), however, the number of studies within grape juice and pomegranate juice interventions (n ranged from 3 to 4) were too few to perform subgroup analysis. The high heterogeneity could be explained by the different populations and regions and participant characteristics at baseline within these few studies. Physical activity level has been considered as cofactor, but no adjustments for physical activity level have been applied among the included studies in this review.

There is limited study data under some types of interventions investigating all risk factors (i.e. grape powder and cherry juice) to be meta-analysed; and even though studies supplementing cranberry juice have shown an significant effect, they are not accompanied by improvements to other risk factors and are limited to relatively small sample sizes within 2 studies, so the implications of our results should be treated with caution. Heterogeneities presented in our results, however, were explored by subgroup analyses of different intervention subgroups, due to the limited number of studies under each participants characteristic and country region, we were unable to further compare among different baseline-characterised subjects (i.e. physical activity, gender), regions (i.e. western and other countries) and juice qualities.

## Conclusion

This review has highlighted a scarcity of intervention studies aimed at improving endothelial function and cardiovascular health by consuming berries, citrus and cherries in different forms such as freeze-dried and powdered fruit or as fruit juice. The quantitative analysis led us to further explore the potential of various berries, cherries and citrus-based interventions to improve endothelial function and cardiovascular health. There is a potential for berries in juiced forms to benefit cardio-health, however, these are only suggestive and raised from non-substantial evidence from a few studies within each intervention type. Inconsistent evidence was reported considering results from our analysis along with other reviews regarding the effect of fruit juice on CVD risk factors. More research supplementing summarised interventions in this review is warranted to reinforce the evidence and to further substantiate the health benefits of specific fruit-based interventions.

## Electronic supplementary material

Below is the link to the electronic supplementary material.

Supplementary material 1 (PDF 4309 kb)

Supplementary material 2 (DOCX 12 kb)

Supplementary material 3 (PDF 63 kb)

Supplementary material 4 (DOCX 11 kb)

Supplementary material 5 (DOCX 130 kb)

Supplementary material 6 (DOCX 16 kb)

Supplementary material 7 (PDF 51 kb)
