# Correction to: Effects of chronic consumption of specific fruit (berries, citrus and cherries) on CVD risk factors: a systematic review and meta‑analysis of randomised controlled trials

**DOI:** 10.1007/s00394-020-02456-1

**Published:** 2021-01-23

**Authors:** Yueyue Wang, Jose Lara Gallegos, Crystal Haskell-Ramsay, John K. Lodge

**Affiliations:** 1 Department of Applied Sciences, Faculty of Health and Life Sciences, Northumbria University, EBD223 Ellison Building, Newcastle upon Tyne, NE1 8ST UK; 2 Department of Psychology, Faculty of Health and Life Sciences, Northumbria University, Newcastle-upon-Tyne, UK

## Correction to: European Journal of Nutrition 10.1007/s00394-020-02299-w

In the original publication, a study supplementing orange juice by Morand, et al., 2011 (France) was incorrectly reported for the diastolic blood pressure (DBP) outcome. We originally reported no improvement in DBP as results were reported as least square means, when actually Morand and investigators found a significant improvement in DBP by orange juice compared to placebo. The study should have been reported in Table 1 as “significant improvement compared to the control”, and therefore there should be 11 interventions in the review reporting improvements on blood pressures. We include here an updated forest plot of the citrus juice group, that includes the correct findings of the Morand study investigating the outcome of DBP (Fig. 6). In our updated meta-analysis there was no significant improvement in DBP by the citrus juice interventions compared to the control. The *I*^2^ test suggested significant substantial heterogeneities for citrus juice group investigating the effects on DBP (*I*^2^ = 83*%, P* < 0.01) (Fig. 6). The sensitivity analysis also suggested no effect of grapefruit concentrate juice in the citrus juice group on the result of DBP (Supplemental Table 6). We apologise for this error in misreporting the study results of Morand and investigators.

Please find the corrected Table 1 and Fig. 6 below.

**Table 1:**
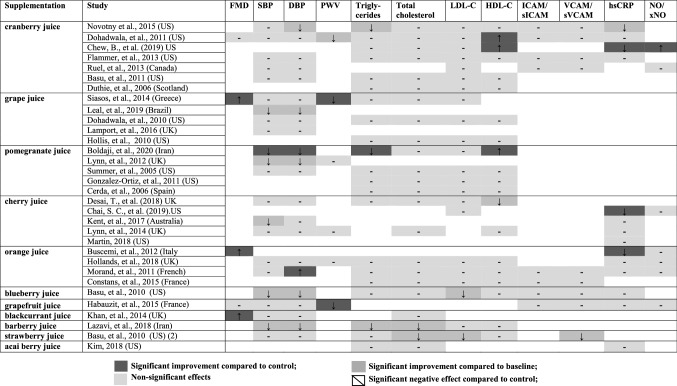
Qualitative summarization for fruit juice interventions

**Fig. 6:**
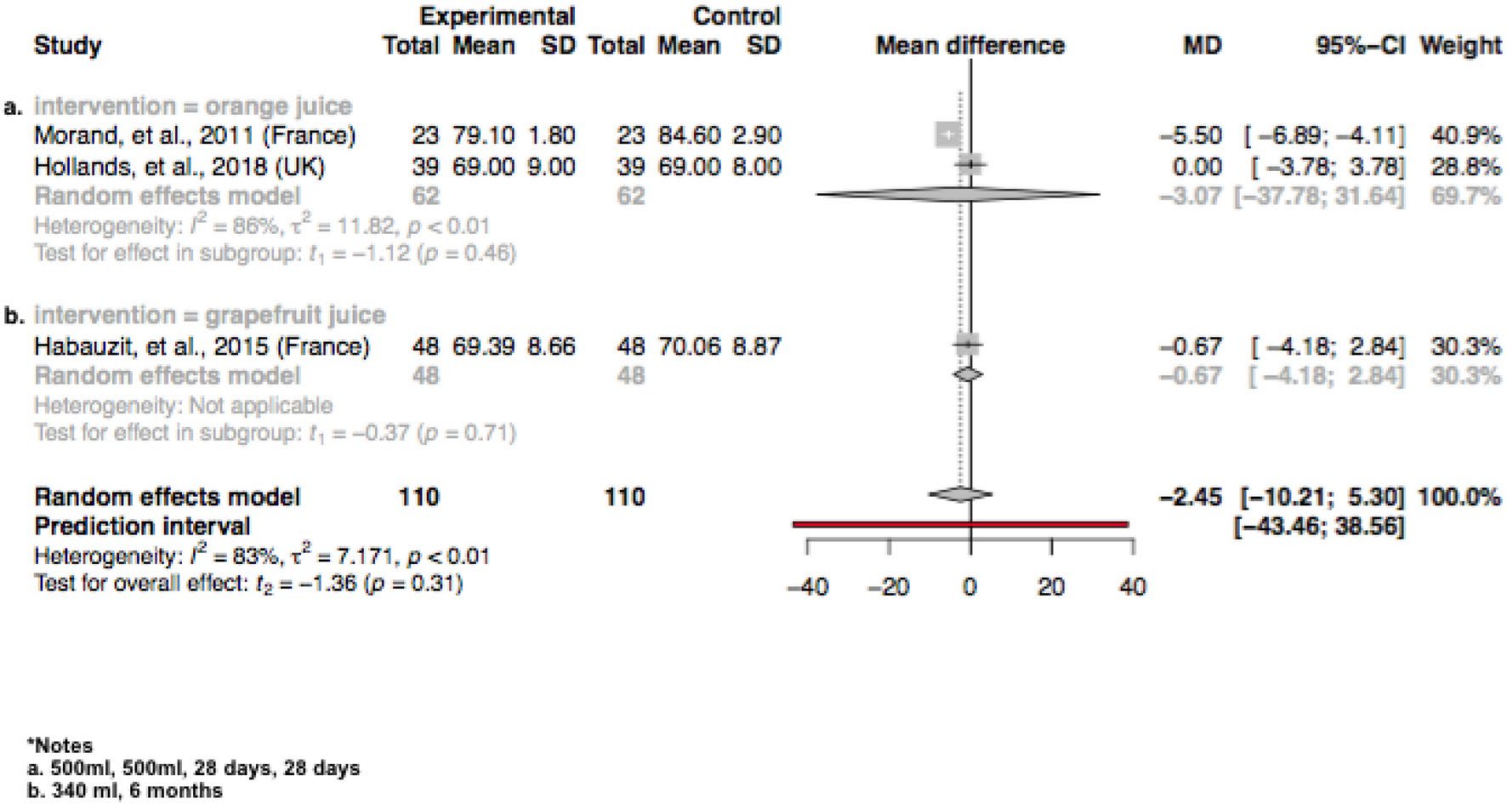
The effect of citrus interventions including **a** orange juice and **b** grapefruit juice assessing DBP

