# Supplementary material for: Effects of chronic consumption of specific fruit (berries, citrus and cherries) on CVD risk factors: a systematic review and meta-analysis of randomised controlled trials
Source: Eur J Nutr. 2020 Jun 13;60(2):615–39. doi: 10.1007/s00394-020-02299-w (PMC7900084; doi:10.1007/s00394-020-02299-w)
Supplement: Supplementary file 1 — Supplementary material 1 (PDF 4309 kb) [file 394_2020_2299_MOESM1_ESM.pdf]

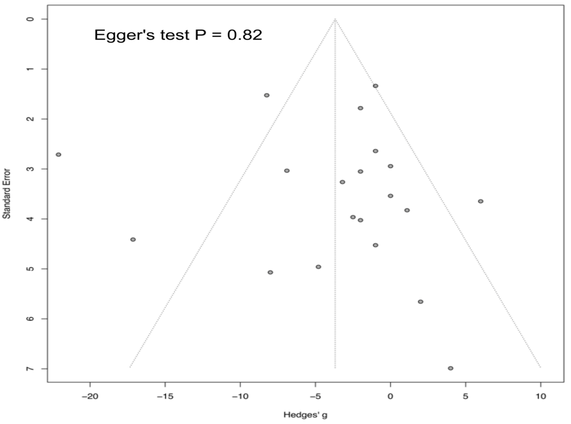

a. Funnel plot of investigated effect of berry group on SBP

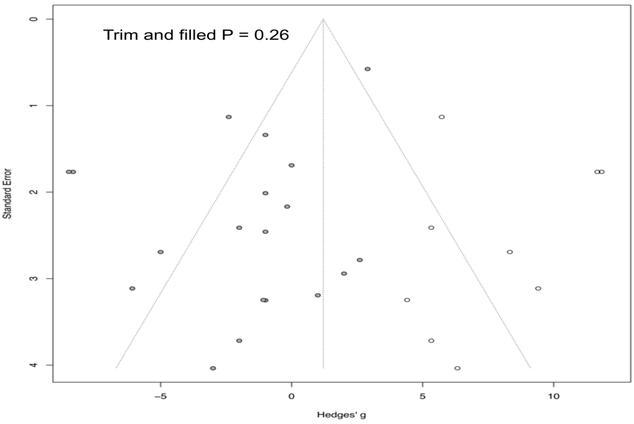

b. Funnel plot of investigated effect of berry group on DBP

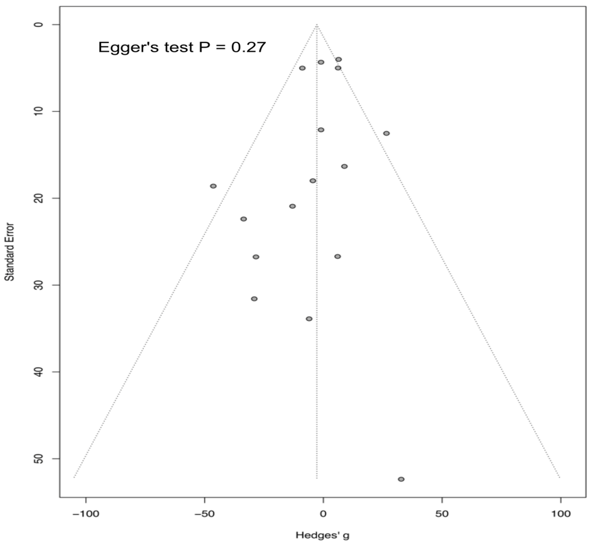

c. Funnel plot of investigated effect of berry group on TAG

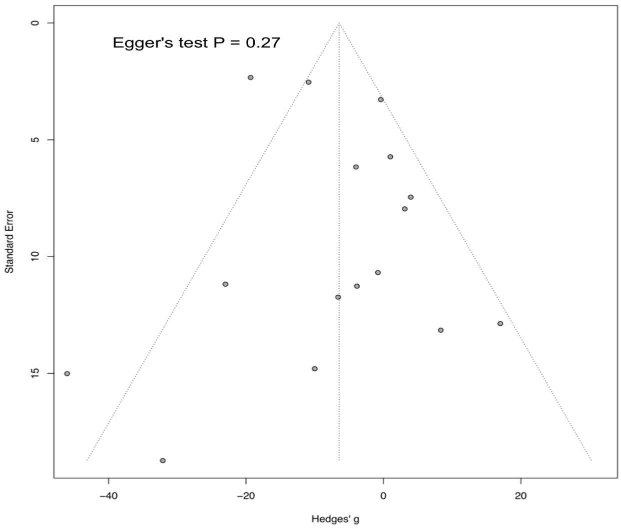

d. Funnel plot of investigated effect of berry group on TC

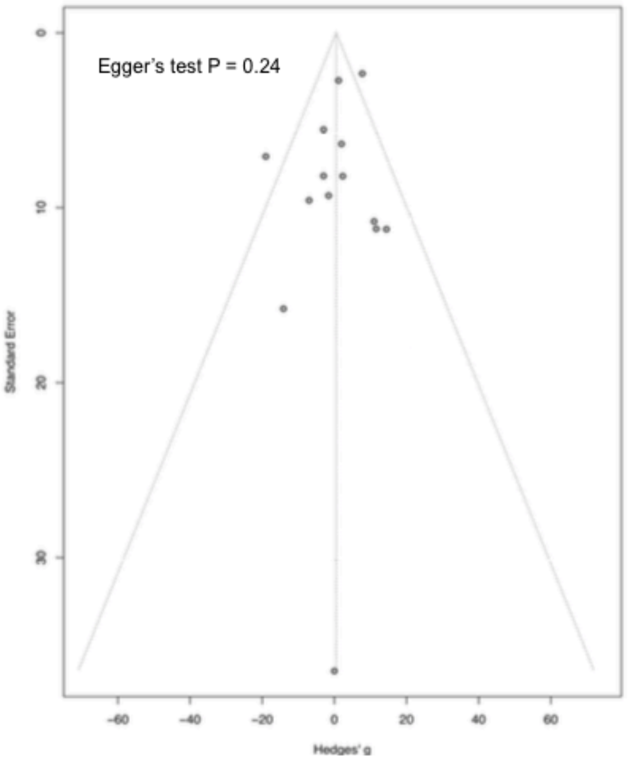

e. Funnel plot of investigated effect of berry group on LDL-C

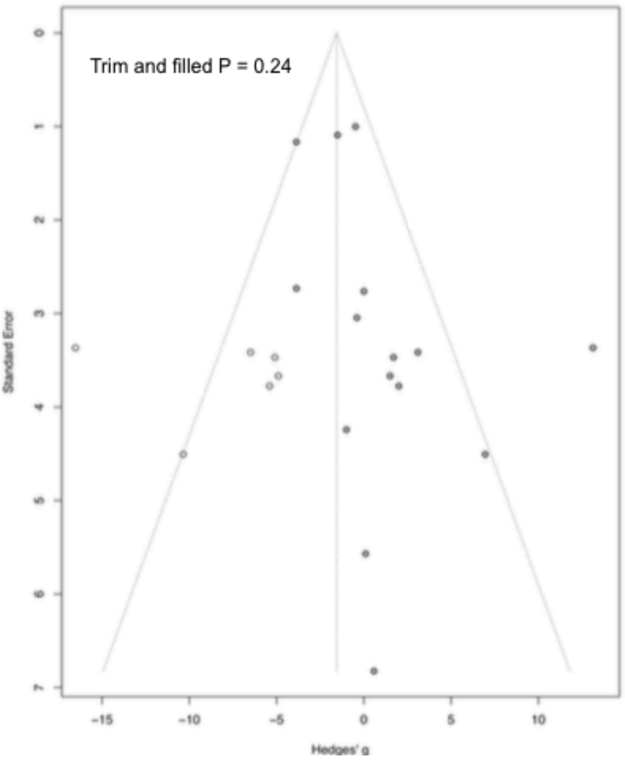

f. Funnel plot of investigated effect of berry group on HDL-C

Supplemental Figure 1. Funnel plots of the effects of berries interventions on a.SBP, b.DBP, c.TAG, d.TC, e. LDL-C, f. HDL-C

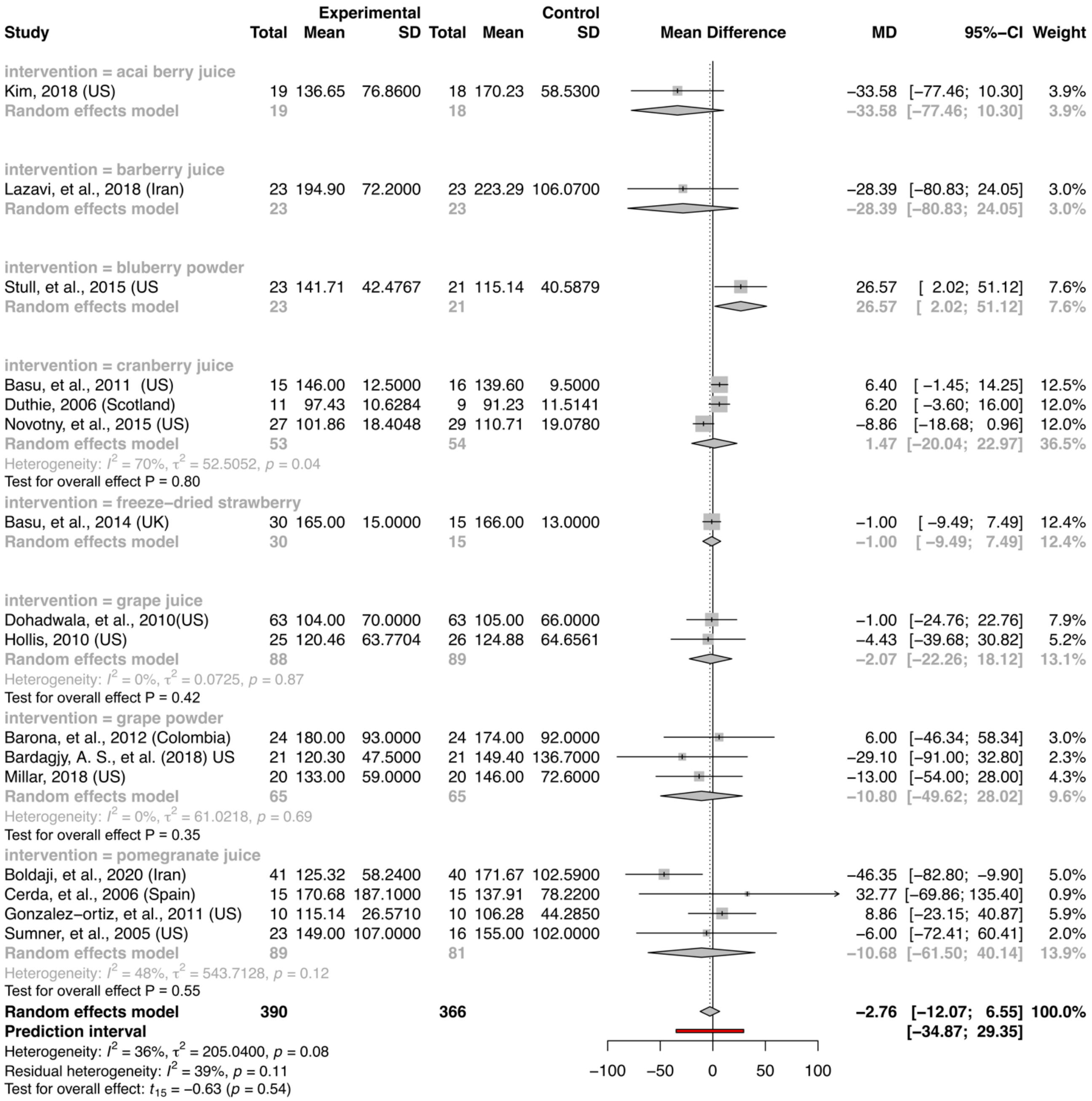

Supplemental Figure 2. The effect of the berry interventions on TAG

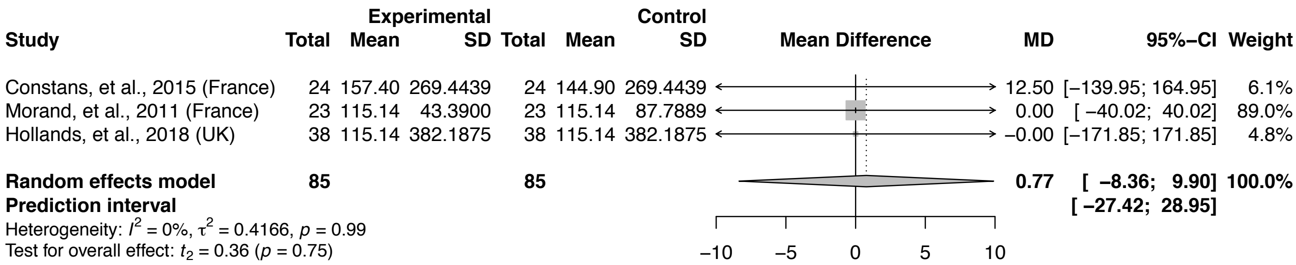

Supplemental Figure 3. The effect of orange juice interventions on TAG

Study	Experimental Total	Experimental Mean	Experimental SD	Control Total	Control Mean	Control SD	MD	95%-CI	Weight
**intervention = acai berry juice**									
Kim, 2018 (US)	19	195.12	62.1400	18	227.23	51.5500	−32.11	[−68.82; 4.60]	2.9%
Random effects model	19			18			−32.11	[−68.82; 4.60]	2.9%
**intervention = barberry juice**									
Lazavi, et al., 2018 (Iran)	23	203.10	47.9700	23	249.14	53.6700	−46.04	[−75.46; −16.62]	3.9%
Random effects model	23			23			−46.04	[−75.46; −16.62]	3.9%
**intervention = bluberry powder**									
Stull, et al., 2015 (US	23	174.02	7.7340	21	193.35	7.7340	−19.34	[−23.91; −14.76]	9.6%
Random effects model	23			21			−19.34	[−23.91; −14.76]	9.6%
**intervention = cranberry juice**									
Basu, et al., 2011 (US)	15	196.40	30.3000	16	203.00	35.0000	−6.60	[−29.61; 16.41]	5.1%
Duthie, 2006 (Scotland)	11	189.34	28.1956	9	172.33	28.9800	17.00	[−8.22; 42.22]	4.6%
Novotny, et al., 2015 (US)	27	196.29	12.0480	29	196.68	12.4846	−0.39	[−6.81; 6.04]	9.3%
Random effects model	53			54			1.55	[−22.29; 25.39]	19.1%
Heterogeneity: $I^2 = 3\%$, $\tau^2 = 60.4074$, $p = 0.35$									
Test for overall effect P = 0.81									
**intervention = freeze−dried strawberry**									
Basu, et al., 2014 (UK)	30	181.00	27.3861	29	204.00	53.8516	−23.00	[−44.91; −1.09]	5.3%
Random effects model	30			29			−23.00	[−44.91; −1.09]	5.3%
**intervention = grape juice**									
Dohadwala, et al., 2010(US)	63	189.00	32.0000	63	193.00	37.0000	−4.00	[−16.08; 8.08]	7.9%
Hollis, 2010 (US)	25	174.40	27.0480	26	171.31	29.7528	3.09	[−12.50; 18.69]	6.9%
Siasos, et al., 2014 (Greece)	26	166.00	23.0000	26	165.00	18.0000	1.00	[−10.23; 12.23]	8.1%
Random effects model	114			115			−0.35	[−9.07; 8.38]	23.0%
Heterogeneity: $I^2 = 0\%$, $\tau^2 = 2.1803$, $p = 0.74$									
Test for overall effect P = 0.88									
**intervention = grape powder**									
Bardagjy, A. S., et al. (2018) US	21	201.40	32.4000	21	202.20	36.7000	−0.80	[−21.74; 20.14]	5.6%
Millar, 2018 (US)	20	191.00	46.9000	20	201.00	46.7000	−10.00	[−39.01; 19.01]	4.0%
Random effects model	41			41			−3.99	[−59.63; 51.65]	9.5%
Heterogeneity: $I^2 = 0\%$, $\tau^2 = 4.7700$, $p = 0.61$									
Test for overall effect P = 0.53									
**intervention = pomegranate juice**									
Boldaji, et al., 2020 (Iran)	41	149.82	36.4600	40	145.85	30.4500	3.97	[−10.64; 18.58]	7.2%
Cerda, et al., 2006 (Spain)	15	209.68	39.1000	15	201.34	32.6400	8.34	[−17.44; 34.12]	4.5%
Gonzalez−ortiz, et al., 2011 (US)	10	181.61	27.0480	10	185.47	23.1840	−3.86	[−25.94; 18.22]	5.3%
Sumner, et al., 2005 (US)	21	149.00	6.6000	19	159.90	9.1000	−10.90	[−15.87; −5.93]	9.6%
Random effects model	87			84			−4.31	[−18.06; 9.44]	26.6%
Heterogeneity: $I^2 = 45\%$, $\tau^2 = 36.7060$, $p = 0.14$									
Test for overall effect P = 0.39									
**Random effects model**	**390**			**385**			**−6.45**	**[−13.69; 0.80]**	**100.0%**
**Prediction interval**								**[−33.19; 20.29]**	

Heterogeneity: $I^2 = 71\%$, $\tau^2 = 143.8551$, $p < 0.01$
Residual heterogeneity: $I^2 = 5\%$, $p = 0.39$
Test for overall effect: $t_{15} = -1.90$ ($p = 0.08$)

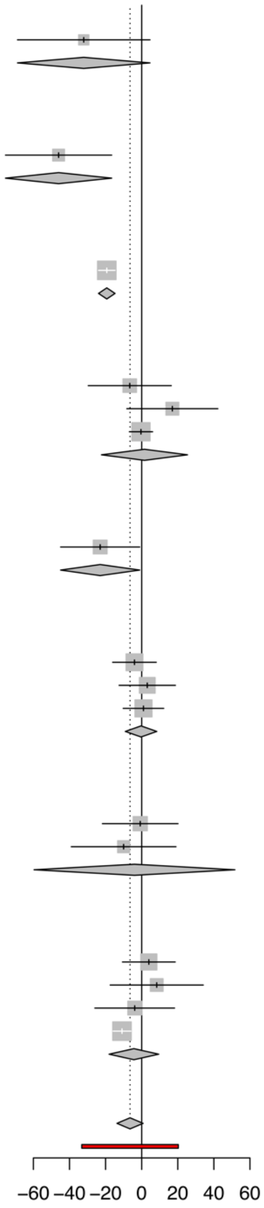

Supplemental Figure 4. The effect of the berry interventions on TAG

Study	Experimental Total	Experimental Mean	Experimental SD	Control Total	Control Mean	Control SD	MD	95%-CI	Weight
Lynn, et al., 2014 (UK)	25	4.22	0.7700	21	4.12	0.6700	0.10	[−0.32; 0.52]	30.7%
Desai, T., et al. (2018) UK	11	2.94	0.2000	11	3.17	0.0600	−0.23	[−0.35; −0.11]	69.3%
**Random effects model**	**36**			**32**			**−0.13**	**[−2.06; 1.80]**	**100.0%**

Heterogeneity: $I^2 = 55\%$, $\tau^2 = 0.0286$, $p = 0.14$
Test for overall effect: $t_1 = -0.85$ ($p = 0.55$)

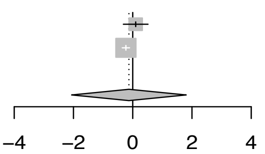

a. The effect of cherry juice interventions on TC

Study	Experimental Total	Experimental Mean	Experimental SD	Control Total	Control Mean	Control SD	MD	95%-CI	Weight
Constans, et al., 2015 (France)	24	249.80	102.8786	24	228.40	102.8786	21.40	[−36.81; 79.61]	7.0%
Morand, et al., 2011 (France)	23	220.42	37.0910	23	212.69	37.0910	7.73	[−13.70; 29.17]	41.1%
Hollands, et al., 2018 (UK)	38	197.22	38.6700	38	201.08	42.5370	−3.87	[−22.14; 14.41]	51.9%
**Random effects model**	**85**			**85**			**2.66**	**[−20.35; 25.68]**	**100.0%**
**Prediction interval**								**[−99.82; 105.14]**	

Heterogeneity: $I^2 = 0\%$, $\tau^2 = 36.4443$, $p = 0.58$
Test for overall effect: $t_2 = 0.50$ ($p = 0.67$)

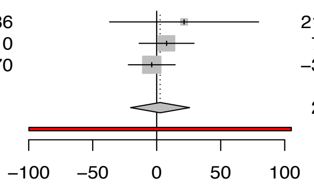

b. The effect of orange juice interventions on TC

Supplemental Figure 5. The effect of cherry and orange juice interventions on TC

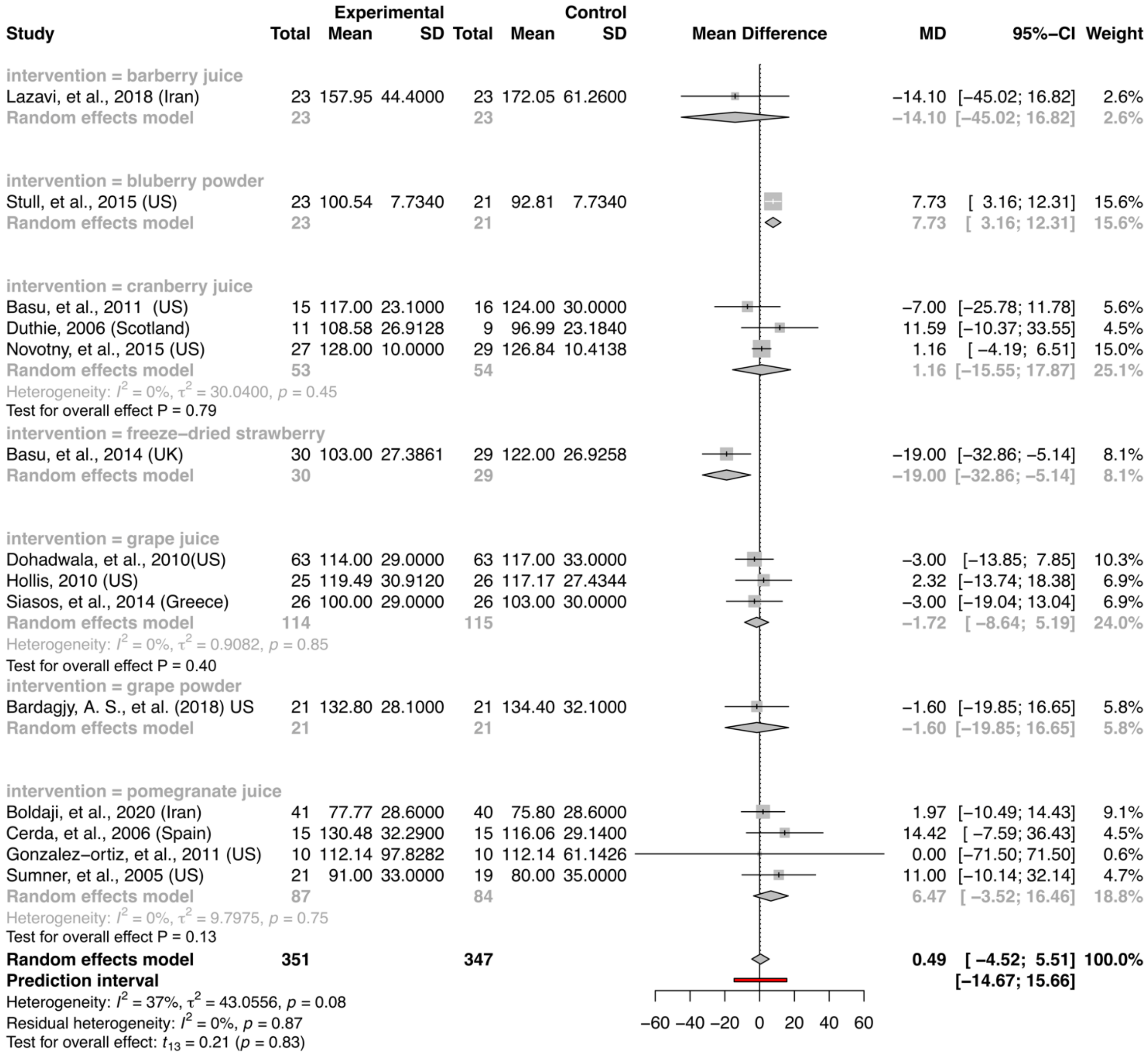

Supplemental Figure 6. The effect of the berry interventions on LDL-C

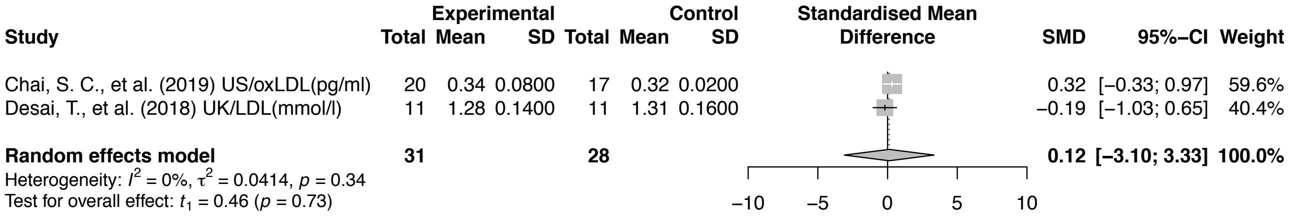

Supplemental Figure 7. The effect of cherry juice interventions on LDL-C

Study	Experimental Total	Experimental Mean	Experimental SD	Control Total	Control Mean	Control SD	MD	95%-CI	Weight
intervention = barberry juice									
Hollis, 2010 (US)	25	54.14	11.2056	26	51.04	13.1376	3.09	[−3.60; 9.79]	6.1%
Lazavi, et al., 2018 (Iran)	23	32.31	3.1200	23	32.79	3.6600	−0.48	[−2.45; 1.49]	11.4%
Random effects model	48			49			0.18	[−17.47; 17.84]	17.6%
Heterogeneity: $I^2 = 1\%$, $\tau^2 = 2.1401$, $p = 0.32$									
Test for overall effect P = 0.92									
intervention = bluberry powder									
Stull, et al., 2015 (US	23	42.54	3.8670	21	46.40	3.8670	−3.87	[−6.15; −1.58]	11.1%
Random effects model	23			21			−3.87	[−6.15; −1.58]	11.1%
intervention = cranberry juice									
Basu, et al., 2011 (US)	15	46.40	8.9000	16	44.70	10.4000	1.70	[−5.10; 8.50]	6.0%
Dohadwala, et al., 2010(US)	63	54.00	15.0000	63	54.00	16.0000	0.00	[−5.42; 5.42]	7.4%
Novotny, et al., 2015 (US)	27	47.56	4.0147	29	49.07	4.1615	−1.51	[−3.65; 0.63]	11.3%
Random effects model	105			108			−0.93	[−4.13; 2.27]	24.7%
Heterogeneity: $I^2 = 0\%$, $\tau^2 = 0.5962$, $p = 0.62$									
Test for overall effect P = 0.34									
intervention = freeze−dried strawberry									
Basu, et al., 2014 (UK)	30	47.00	16.4317	29	48.00	16.1555	−1.00	[−9.32; 7.32]	4.8%
Random effects model	30			29			−1.00	[−9.32; 7.32]	4.8%
intervention = grape juice									
Cerda, et al., 2006 (Spain)	15	56.67	13.7200	15	56.09	22.5900	0.58	[−12.80; 13.96]	2.4%
Gonzalez−ortiz, et al., 2011 (US)	10	42.50	3.8640	10	46.37	7.7280	−3.86	[−9.22; 1.49]	7.5%
Random effects model	25			25			−3.16	[−23.73; 17.40]	9.9%
Heterogeneity: $I^2 = 0\%$, $\tau^2 = 1.5257$, $p = 0.55$									
Test for overall effect P = 0.30									
intervention = grape powder									
Barona, et al., 2012 (Colombia)	24	42.50	10.8000	24	42.90	10.3000	−0.40	[−6.37; 5.57]	6.8%
Millar, 2018 (US)	20	45.10	18.2000	20	45.00	17.0000	0.10	[−10.81; 11.01]	3.3%
Random effects model	44			44			−0.28	[−2.96; 2.39]	10.1%
Heterogeneity: $I^2 = 0\%$, $\tau^2 = 0.0004$, $p = 0.94$									
Test for overall effect P = 0.41									
intervention = pomegranate juice									
Bardagjy, A. S., et al. (2018) US	21	51.30	10.9000	21	49.80	12.8000	1.50	[−5.69; 8.69]	5.7%
Boldaji, et al., 2020 (Iran)	41	52.33	18.4600	40	39.20	11.0100	13.13	[6.53; 19.73]	6.2%
Duthie, 2006 (Scotland)	11	57.18	12.8169	9	50.23	6.9552	6.95	[−1.88; 15.78]	4.4%
Sumner, et al., 2005 (US)	23	48.00	11.0000	16	46.00	12.0000	2.00	[−5.40; 9.40]	5.5%
Random effects model	96			86			6.00	[−2.91; 14.91]	21.8%
Heterogeneity: $I^2 = 58\%$, $\tau^2 = 18.7582$, $p = 0.07$									
Test for overall effect P = 0.12									
**Random effects model**	**371**			**362**			**0.61**	**[−1.74; 2.95]**	**100.0%**
**Prediction interval**								**[−7.01; 8.22]**	
Heterogeneity: $I^2 = 56\%$, $\tau^2 = 11.2379$, $p < 0.01$									
Residual heterogeneity: $I^2 = 16\%$, $p = 0.30$									
Test for overall effect: $t_{14} = 0.56$ ($p = 0.59$)									

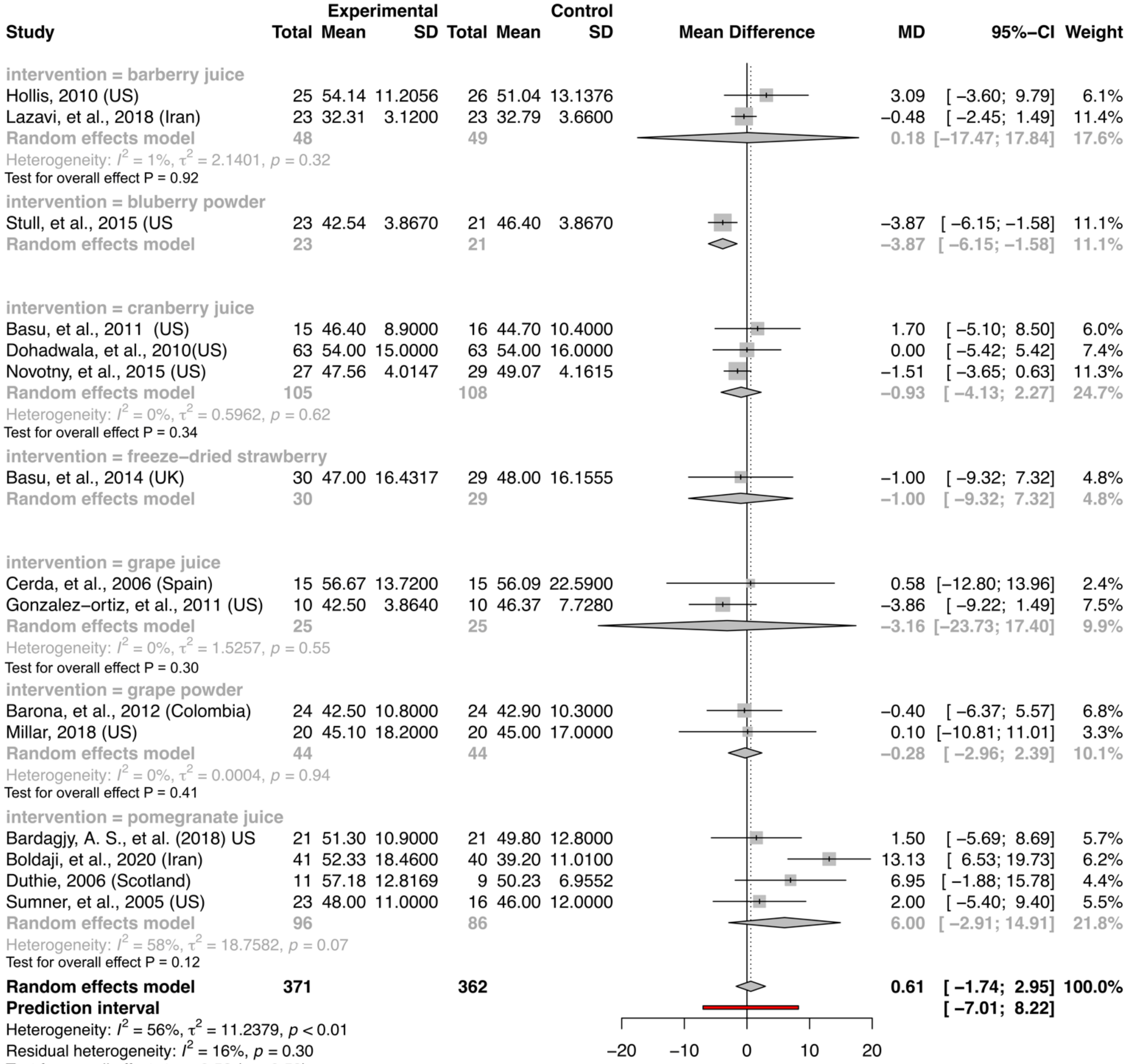

Supplemental Figure 8. The effect of the berry interventions on HDL-C

Study	Experimental Total	Experimental Mean	Experimental SD	Control Total	Control Mean	Control SD	MD	95%-CI	Weight
Constans, et al., 2015 (France)	24	50.04	40.1716	24	47.27	40.1716	2.77	[−19.96; 25.50]	5.3%
Morand, et al., 2011 (France)	23	54.14	18.9440	23	54.14	18.9440	0.00	[−10.95; 10.95]	22.8%
Hollands, et al., 2018 (UK)	38	50.27	11.6010	38	50.27	15.4680	0.00	[−6.15; 6.15]	72.0%
**Random effects model**	**85**			**85**			**0.15**	**[−1.74; 2.03]**	**100.0%**
**Prediction interval**								**[−6.04; 6.34]**	
Heterogeneity: $I^2 = 0\%$, $\tau^2 = 0.0452$, $p = 0.97$									
Test for overall effect: $t_2 = 0.33$ ($p = 0.77$)									

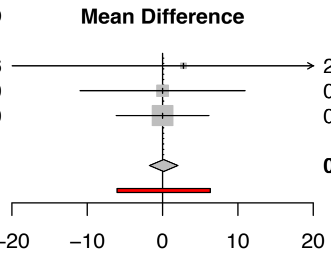

Supplemental Figure 9. The effect of orange juice interventions on HDL-C

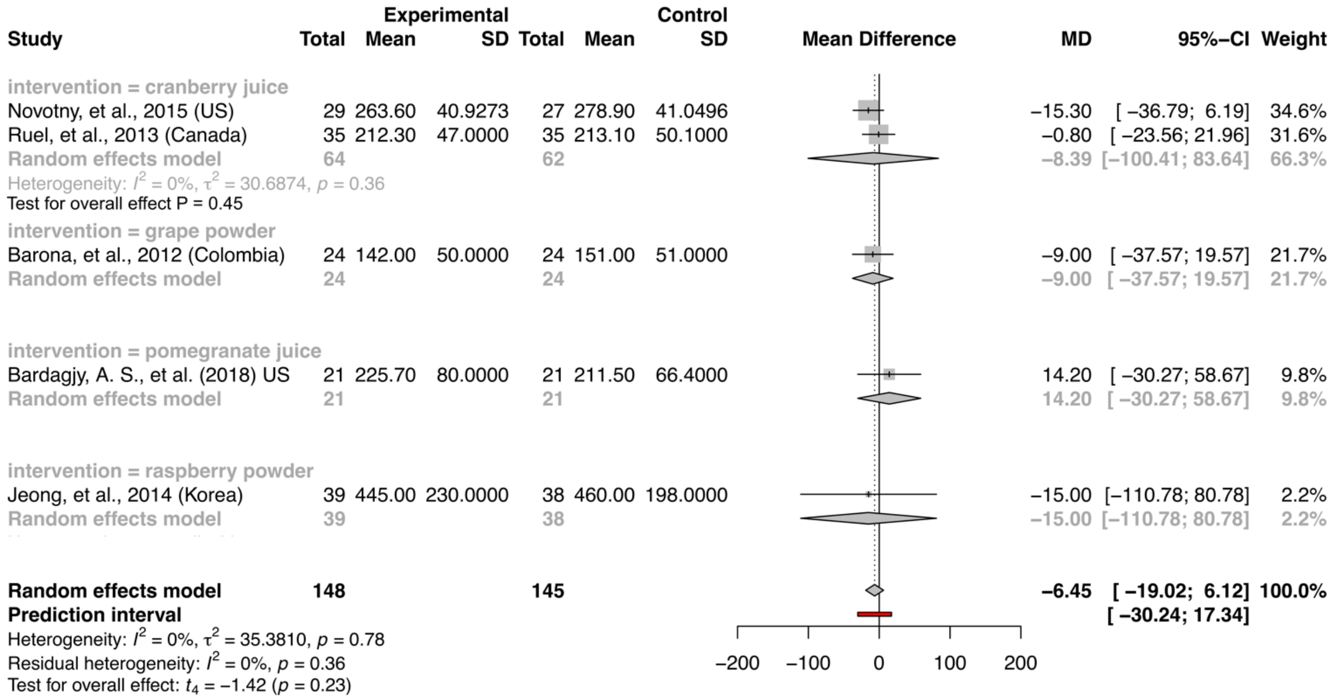

Supplemental Figure 10. The effect of the berry interventions on ICAM

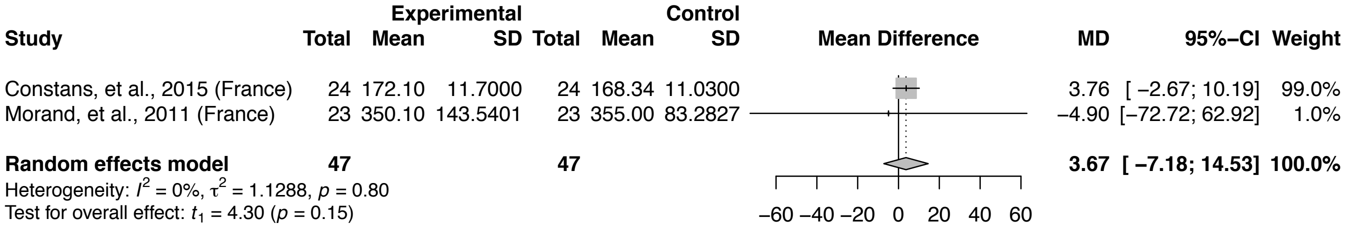

Supplemental Figure 11. The effect of orange juice interventions on ICAM

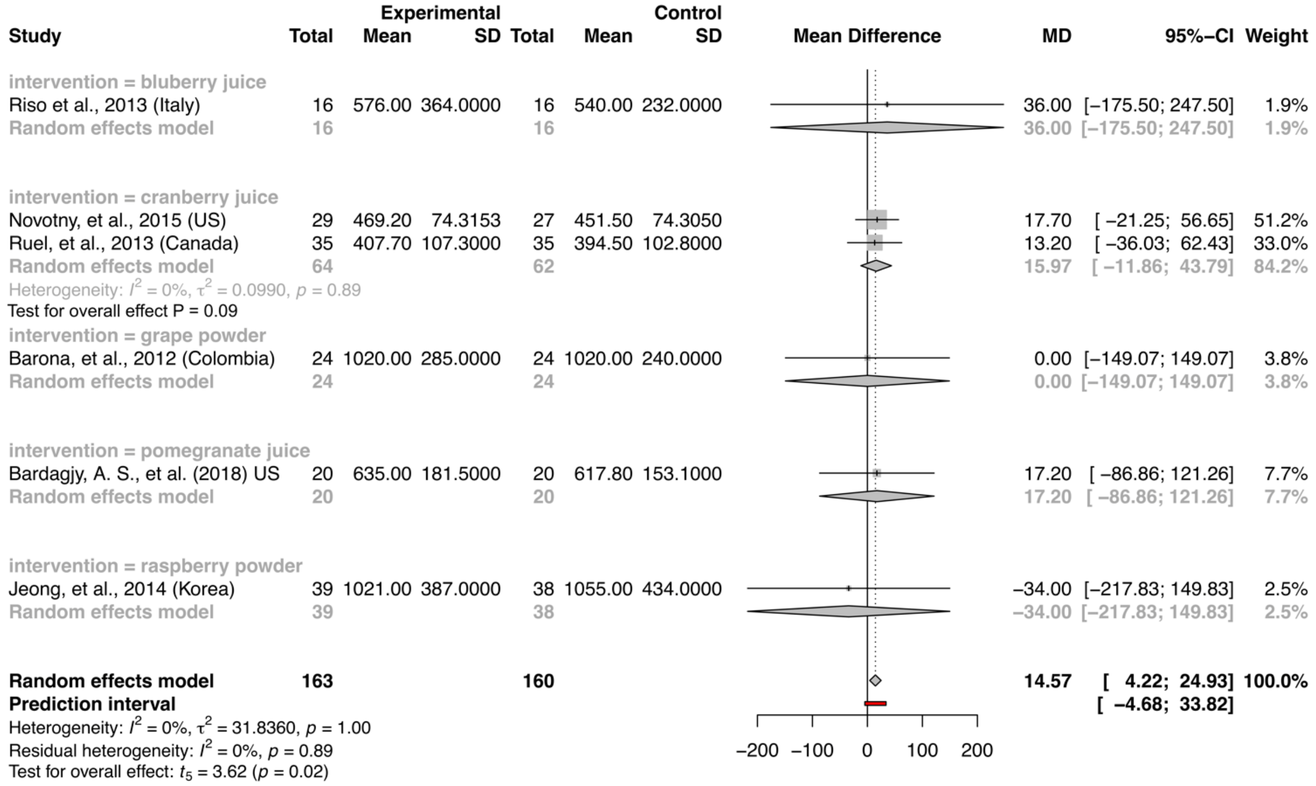

Supplemental Figure 12. The effect of the berry interventions on VCAM

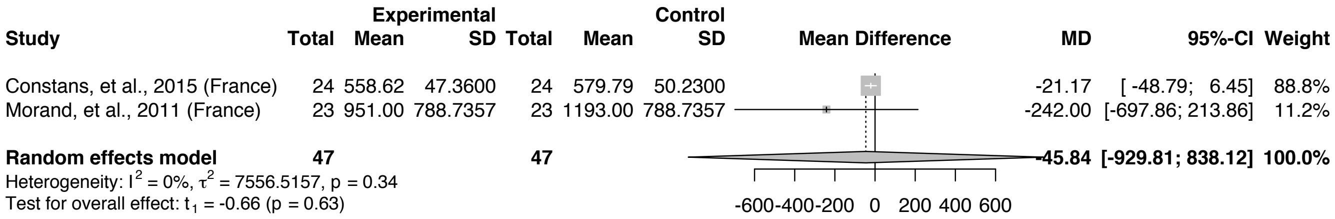

Supplemental Figure 13. The effect of orange juice interventions on VCAM

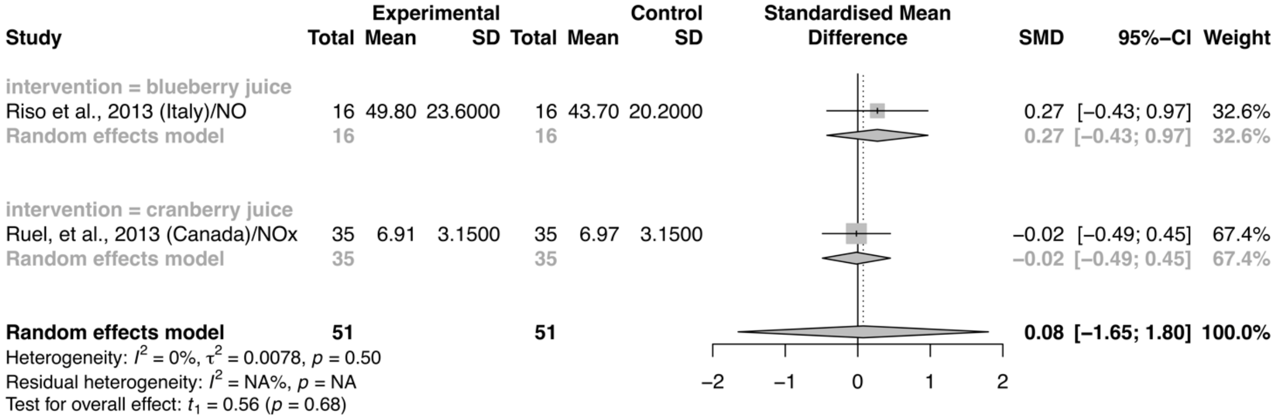

Supplemental Figure 14. The effect of the berry interventions on NO

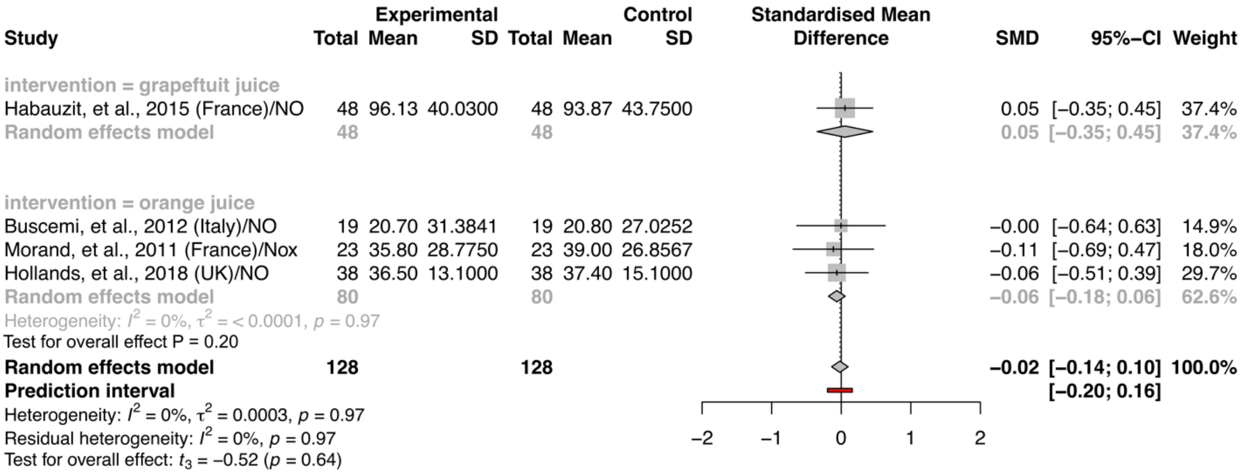

Supplemental Figure 15. The effect of citrus juice interventions on NO

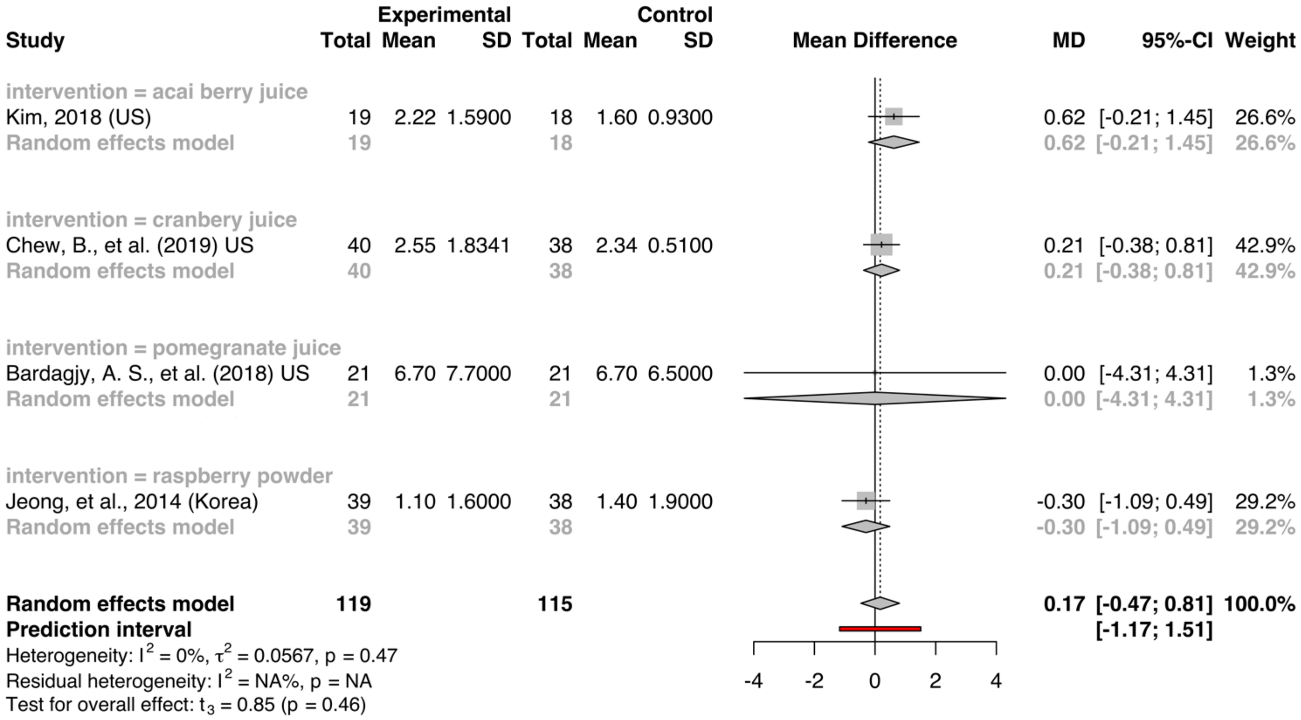

Supplemental Figure 16. The effect of the berry interventions on hsCRP

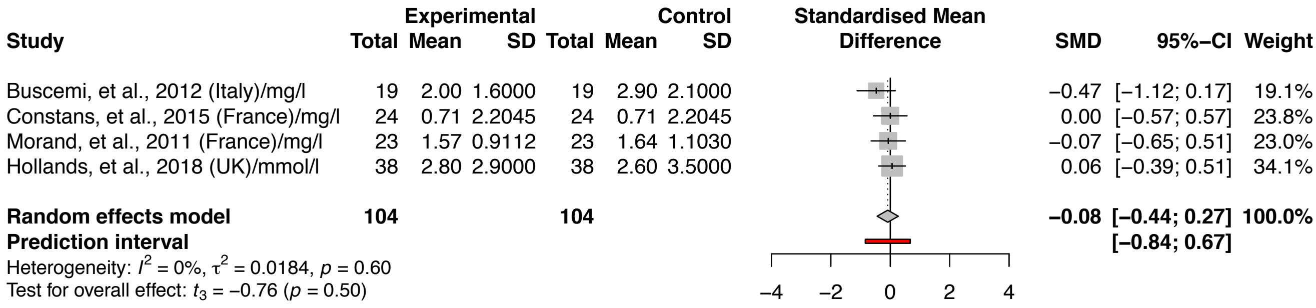

Supplemental Figure 17. The effect of orange juice interventions on hsCRP
