# Supplementary material for: Effects of chronic consumption of specific fruit (berries, citrus and cherries) on CVD risk factors: a systematic review and meta-analysis of randomised controlled trials
Source: Eur J Nutr. 2020 Jun 13;60(2):615–39. doi: 10.1007/s00394-020-02299-w (PMC7900084; doi:10.1007/s00394-020-02299-w)
Supplement: Supplementary file 2 — Supplementary material 2 (DOCX 12 kb) [file 394_2020_2299_MOESM2_ESM.docx]

**Supplemental Table 1. PICOS framework**

| **Population** | Adult subjects ≥ 18 years of age |
| --- | --- |
| **Intervention** | Interventional studies providing or promoting citrus, cherry, or berry fruits or their juices or freeze-dried powdered forms (i.e. their juices) to be consumed. Intervention length is at least 1 week |
| **Comparator** | Control groups without components of citrus, cherry, or berry fruits, likely isoenergetic placebo group |
| **Outcome** | The primary outcomes are systolic and diastolic blood pressure and the endothelial function assessed by flow-mediated dilation and pulse wave velocity; the secondary pitcomes are the CVD risk factors including circulating fatty acids Triglycerides/TAGs and lipoproteins TC, LDL-L and HDL-C; inflammatory biomarkers such as C-reactive protein/CRP, Nitric Oxide/NO, Intercellular Adhesion Molecules/ICAMs and Vascular Adhesion Molecules/VCAMs were also explored |
| **Study design** | Randomised controlled trials (RCTs) |
