# Supplementary material for: Effects of chronic consumption of specific fruit (berries, citrus and cherries) on CVD risk factors: a systematic review and meta-analysis of randomised controlled trials
Source: Eur J Nutr. 2020 Jun 13;60(2):615–39. doi: 10.1007/s00394-020-02299-w (PMC7900084; doi:10.1007/s00394-020-02299-w)
Supplement: Supplementary file 4 — Supplementary material 4 (DOCX 11 kb) [file 394_2020_2299_MOESM4_ESM.docx]

(Fruit OR citrus OR orange OR berry OR berries OR grape OR blueberry OR blueberries OR blackberry OR blackberries OR raspberry OR raspberries OR cranberry OR cranberries OR cherry OR cherries) AND ("endothelial function" OR "vascular function" OR "vascular risk factors" OR hypertension OR "blood pressure" OR BP OR "pulse wave velocity" OR PWV OR "flow-mediated dilation" OR FMD OR lipid OR cholesterol OR LDL OR HDL OR triglyceride OR biomarkers OR inflammatory OR Nitric Oxide OR NO OR ICAM OR VCAM OR CRP) AND (trial OR intervention)
