# Supplementary material for: Effects of chronic consumption of specific fruit (berries, citrus and cherries) on CVD risk factors: a systematic review and meta-analysis of randomised controlled trials
Source: Eur J Nutr. 2020 Jun 13;60(2):615–39. doi: 10.1007/s00394-020-02299-w (PMC7900084; doi:10.1007/s00394-020-02299-w)
Supplement: Supplementary file 5 — Supplementary material 5 (DOCX 130 kb) [file 394_2020_2299_MOESM5_ESM.docx]

**Supplemental Table 4. Excluded reference list for full-text eligibility**

| **Excluded references** | **Exclusion reason** |
| --- | --- |
| Vaisman N, Niv E (2015) Daily consumption of red grape cell powder in a dietary dose improves cardiovascular parameters: a double blind, placebo-controlled, randomized study. *International journal of food sciences and nutrition* **66**, 342-349. | **Treatment is not relevant** |
| McCall DO, McGartland CP, McKinley MC *et al.* (2009) Dietary intake of fruits and vegetables improves microvascular function in hypertensive subjects in a dose-dependent manner. *Circulation* **119**, 2153-2160. | **Vegetable and fruits treatment source** |
| Berry SE, Mulla UZ, Chowienczyk PJ *et al.* (2010) Increased potassium intake from fruit and vegetables or supplements does not lower blood pressure or improve vascular function in UK men and women with early hypertension: a randomised controlled trial. *The British journal of nutrition* **104**, 1839-1847. |  |
| George TW, Waroonphan S, Niwat C *et al.* (2013) Effects of acute consumption of a fruit and vegetable puree-based drink on vasodilation and oxidative status. *The British journal of nutrition* **109**, 1442-1452. |  |
| Macready AL, George TW, Chong MF *et al.* (2014) Flavonoid-rich fruit and vegetables improve microvascular reactivity and inflammatory status in men at risk of cardiovascular disease-FLAVURS: a randomized controlled trial(1-5). *American Journal of Clinical Nutrition* **99**, 479-489. |  |
| Ali A, Yazaki Y, Njike VY *et al.* (2011) Effect of fruit and vegetable concentrates on endothelial function in metabolic syndrome: a randomized controlled trial. *Nutrition journal* **10**, 72. |  |
| Noad RL, Rooney C, McCall D *et al.* (2016) Beneficial effect of a polyphenol-rich diet on cardiovascular risk: a randomised control trial. *Heart (British Cardiac Society)* **102**, 1371-1379. |  |
| Erlund I, Koli R, Alfthan G *et al.* (2008) Favorable effects of berry consumption on platelet function, blood pressure, and HDL cholesterol (every other day: mixed berries+ berry smoothies ). *American Journal of Clinical Nutrition* **87**, 323-331. | **Mixed berries treatment source** |
| Loo B-M, Erlund I, Koli R *et al.* (2016) Consumption of chokeberry (Aronia mitschurinii) products modestly lowered blood pressure and reduced low-grade inflammation in patients with mildly elevated blood pressure. *Nutrition Research* **36**, 1222-1230. |  |
| Dalgård C, Nielsen F, Morrow JD *et al.* (2009) Supplementation with orange and blackcurrant juice, but not vitamin E, improves inflammatory markers in patients with peripheral arterial disease (OJ+Blackcurrant juice+VE). *British Journal of Nutrition* **101**, 263-269. | **Orange & Blackcurrant juice as treatment juice** |
| Asgary S, Keshvari M, Afshani MR *et al.* (2014) Effect of fresh orange juice intake on physiological characteristics in healthy volunteers. *ISRN nutrition* **2014**, 405867. | **Fruit juice as control** |
| Folts JD (2002) Effects of red wine and purple grape juice flavonoids on platelet and endothelial function. *Free Radical Biology and Medicine* **33**, S304-S304. | **Red wine as control** |
| Greyling A, Bruno RM, Draijer R *et al.* (2016) Effects of wine and grape polyphenols on blood pressure, endothelial function and sympathetic nervous system activity in treated hypertensive subjects. *Journal of Functional Foods* **27**, 448-460. |  |
| Auclair S, Chironi G, Milenkovic D *et al.* (2010) The regular consumption of a polyphenol-rich apple does not influence endothelial function: a randomised double-blind trial in hypercholesterolemic adults. *European journal of clinical nutrition* **64**, 1158-1165.  16. | **Apple polyphenols as control** |
| Hashemi M, Kelishadi R, Hashemipour M *et al.* (2010) Acute and long-term effects of grape and pomegranate juice consumption on vascular reactivity in paediatric metabolic syndrome. *Cardiology in the young* **20**, 73-77. | **paediatric patients as subjects** |
| Kelishadi R, Gidding SS, Hashemi M *et al.* (2011) Acute and long term effects of grape and pomegranate juice consumption on endothelial dysfunction in pediatric metabolic syndrome. *Journal of research in medical sciences : the official journal of Isfahan University of Medical Sciences* **16**, 245-253. |  |
