# Supplementary material for: Effects of chronic consumption of specific fruit (berries, citrus and cherries) on CVD risk factors: a systematic review and meta-analysis of randomised controlled trials
Source: Eur J Nutr. 2020 Jun 13;60(2):615–39. doi: 10.1007/s00394-020-02299-w (PMC7900084; doi:10.1007/s00394-020-02299-w)
Supplement: Supplementary file 6 — Supplementary material 6 (DOCX 16 kb) [file 394_2020_2299_MOESM6_ESM.docx]

**Supplemental Table 5. Risk of bias assessments**

| **(Author/ year) country of origin** | **Jadad Score- Randomisation (1 or 0)** | **Jadad Score- blinding (1 or 0)** | **Jada score -withdrawals (1 or 0)** | **Jadad score extra point (1 or 0)** | **Jadad score extra point (1 or 0)** | **Total Jadad score (0 to 5)** |  |  |
| --- | --- | --- | --- | --- | --- | --- | --- | --- |
| **Alquarashi, R. et al. 2016 (UK)** | 1 | 1 | 0 | 1 | 1 | 4 |  |  |
| **Barona, et al. 2012 (Colombia)** | 1 | 1 | 0 | 0 | 0 | 2 |  |  |
| **Buscemi, et al., 2012 (Italy)** | 1 | 1 | 0 | 0 | 0 | 2 |  |  |
| **Constans, et al., 2015 (France)** | 1 | 1 | 0 | 1 | 0 | 3 |  |  |
| **Del Bo, et al., 2013 (Italy)** | 1 | 0 | 1 | 0 | 0 | 2 |  |  |
| **Dohadwala, et al., 2011 (US)** | 1 | 1 | 0 | 1 | 1 | 4 |  |  |
| **Draijer, et al., 2015 (UK)** | 1 | 1 | 1 | 0 | 1 | 4 |  |  |
| **Habauzit, et al., 2015 (France)** | 1 | 1 | 0 | 1 | 1 | 4 |  |  |
| **Hampton, et al., 2010 (UK)** | 1 | 1 | 1 | 0 | 1 | 4 |  |  |
| **Jin, et al., 2011 (UK)** | 1 | 1 | 1 | 0 | 0 | 3 |  |  |
| **Keane, 2016 (UK)** | 1 | 1 | 1 | 0 | 0 | 3 |  |  |
| **Kean et al., 2016 (UK) (2)** | 1 | 1 | 0 | 0 | 0 | 2 |  |  |
| **Lamport, et al., 2016 (UK)** | 1 | 1 | 0 | 1 | 1 | 4 |  |  |
| **Lamport et al., 2016 (2) (UK)** | 1 | 1 | 0 | 1 | 0 | 3 |  |  |
| **Morand, et al., 2011 (French)** | 1 | 1 | 1 | 1 | 1 | 5 |  |  |
| **Rendeiro, et al., 2017 (UK)** | 1 | 1 | 1 | 1 | 1 | 5 |  |  |
| **Riso et al., 2013 (Italy)** | 1 | 0 | 0 | 1 | 0 | 2 |  |  |
| **Rodriguez. Et al., 2013(UK)** | 1 | 1 | 0 | 1 | 1 | 4 |  |  |
| **Ruel, et al., 2013 (Canada)** | 1 | 1 | 1 | 0 | 0 | 3 |  |  |
| **Schaer et al., 2015 (US)** | 1 | 1 | 0 | 1 | 1 | 4 |  |  |
| **Siasos, et al., 2014 (Greece)** | 1 | 1 | 1 | 0 | 1 | 4 |  |  |
| **Willems, et al., 2015 (UK)** | 1 | 1 | 0 | 0 | 0 | 2 |  |  |
| **Basu, A. et al. 2014 (UK)** | 1 | 0 | 1 | 0 | 0 | 2 |  |  |
| **Basu, et al., 2010 (US)** | 1 | 1 | 0 | 0 | 0 | 2 |  |  |
| **Basu, et al., 2010 (US)** | 1 | 1 | 0 | 0 | 1 | 3 |  |  |
| **Basu, et al., 2011 (US)** | 1 | 1 | 0 | 0 | 1 | 3 |  |  |
| **Cerda 2006a (Spain)** | 1 | 1 | 1 | 0 | 0 | 3 |  |  |
| **Duthie 2006b (Scotland)** | 1 | 0 | 1 | 0 | 0 | 2 |  |  |
| **Del Bo, et al., 2014 (Italy)** | 1 | 0 | 1 | 1 | 0 | 3 |  |  |
| **Dow, et al., 2013 (US)** | 1 | 0 | 0 | 0 | 0 | 1 |  |  |
| **Flammer, et al., 2013 (US)** | 1 | 1 | 0 | 0 | 1 | 3 |  |  |
| **Gonzalez-Ortiz 2011b (US)** | 1 | 1 | 1 | 0 | 0 | 3 |  |  |
| **Hollis 2010a (US)** | 1 | 1 | 1 | 0 | 0 | 3 |  |  |
| **Jeong, et al., 2016 (Korea)** | 1 | 1 | 0 | 1 | 0 | 3 |  |  |
| **Jeong, et al., 2016 (Korea) (2)** | 1 | 1 | 0 | 1 | 0 | 3 |  |  |
| **Jeong, et al., 2014(Korea)** | 1 | 1 | 0 | 0 | 1 | 3 |  |  |
| **Johnson, et al., 2015 (US)** | 1 | 1 | 0 | 1 | 0 | 3 |  |  |
| **Kent, et al., 2017 (Australia)** | 1 | 1 | 0 | 1 | 1 | 4 |  |  |
| **Khan, et al., 2014 (UK)** | 1 | 1 | 0 | 1 | 0 | 3 |  |  |
| **Lekakis, et al., 2005 (Greece)** | 1 | 1 | 1 | 0 | 1 | 4 |  |  |
| **Lynn, et al., 2012 (UK)** | 1 | 0 | 1 | 1 | 0 | 3 |  |  |
| **Lynn, et al., 2014 (UK)** | 1 | 0 | 0 | 1 | 0 | 2 |  |  |
| **McAnulty, et al., 2014 (US)** | 1 | 0 | 1 | 0 | 0 | 2 |  |  |
| **Murkovic, et al., 2004b Australia** | 1 | 1 | 1 | 0 | 0 | 3 |  |  |
| **Novotny et al., 2015 (US)** | 1 | 1 | 0 | 1 | 1 | 4 |  |  |
| **Park, et al., 2009 (Japan)** | 1 | 1 | 1 | 0 | 0 | 3 |  |  |
| **Rodriguez. et al., 2016 (UK)** | 1 | 1 | 0 | 1 | 1 | 4 |  |  |
| **Sumner, et al., 2005 (US)** | 1 | 1 | 0 | 1 | 1 | 4 |  |  |
| **Stull, et al., 2015 (US)** | 1 | 1 | 0 | 1 | 1 | 4 |  |  |
