# Supplementary material for: Effects of chronic consumption of specific fruit (berries, citrus and cherries) on CVD risk factors: a systematic review and meta-analysis of randomised controlled trials
Source: Eur J Nutr. 2020 Jun 13;60(2):615–39. doi: 10.1007/s00394-020-02299-w (PMC7900084; doi:10.1007/s00394-020-02299-w)
Supplement: Supplementary file 7 — Supplementary material 7 (PDF 51 kb) [file 394_2020_2299_MOESM7_ESM.pdf]

Supplemental Table 6. Sensitivity analysis of studies with and without juice concentrate supplementation.

	Cherry juice assessing SBP				Cherry juice assessing DBP				Citrus juice assessing SBP			
	N	Mean difference (95% CI)	P for overall effect	P for heterogeneity	N	Mean difference (95% CI)	P for overall effect	P for heterogeneity	N	Mean difference (95% CI)	P for overall effect	P for heterogeneity
All studies	2	−3.11 [−4.06; −2.15]	0.02	0.98	2	−0.51 [−4.45; 3.43]	0.35	0.9	3	0.83 [-6.14; 7.81]	0.66	0.52
Without juice concentrate studies	1	−3.00 [−13.03; 7.03]	NA	NA	1	0.00 [−8.36; 8.36]	NA	NA	2	0.76 [-36.61; 38.13]	0.84	0.26
